# A Chemically Defined Medium That Supports Mycotoxin Production by Stachybotrys chartarum Enabled Analysis of the Impact of Nitrogen and Carbon Sources on the Biosynthesis of Macrocyclic Trichothecenes and Stachybotrylactam

**DOI:** 10.1128/aem.00163-23

**Published:** 2023-06-20

**Authors:** Katharina Tribelhorn, Magdalena Twarużek, Robert Kosicki, Reinhard K. Straubinger, Frank Ebel, Sebastian Ulrich

**Affiliations:** a Chair of Bacteriology and Mycology, Department of Veterinary Sciences, Faculty of Veterinary Medicine, Institute for Infectious Diseases and Zoonosis, LMU Munich, Munich, Germany; b Department of Physiology and Toxicology, Faculty of Biological Sciences, Kazimierz Wielki University, Bydgoszcz, Poland; Royal Botanic Gardens

**Keywords:** *Stachybotrys*, nitrogen source, carbon source, nutrients, macrocyclic trichothecenes, stachybotrylactam, LC-MS/MS

## Abstract

Stachybotrys chartarum (*Hypocreales*, *Ascomycota*) is a toxigenic fungus that is frequently isolated from water-damaged buildings or improperly stored feed. The secondary metabolites formed by this mold have been associated with health problems in humans and animals. Several authors have studied the influence of environmental conditions on the production of mycotoxins, but these studies focused on undefined or complex substrates, such as building materials and media that impeded investigations of the influence of specific nutrients. In this study, a chemically defined cultivation medium was used to investigate the impact of several nitrogen and carbon sources on growth of *S. chartarum* and its production of macrocyclic trichothecenes (MTs) and stachybotrylactam (STLAC). Increasing concentrations of sodium nitrate were found to positively affect mycelial growth, the level of sporulation, and MT production, while ammonium nitrate and ammonium chloride had an inhibitory effect. Potato starch was the superior and most reliable carbon source tested. Additionally, we observed that the level of sporulation was correlated with the production of MTs but not with that of STLAC. In this study, we provide a chemically well-defined cultivation medium suitable for standardized *in vitro* testing of the capacity of *S. chartarum* isolates to produce macrocyclic trichothecenes.

**IMPORTANCE** Macrocyclic trichothecenes (MTs) are highly toxic secondary metabolites that are produced by certain Stachybotrys chartarum strains, which consequently pose a risk for animals and humans. To identify hazardous, toxin-producing strains by analytical means, it is important to grow them under conditions that support MT production. Nutrients determine growth and development and thus the synthesis of secondary metabolites. Complex rich media are commonly used for diagnostics, but batch differences of supplements pose a risk for inconsistent data. We have established a chemically defined medium for *S. chartarum* and used it to analyze the impact of nitrogen and carbon sources. A key finding is that nitrate stimulates MT production, whereas ammonium suppresses it. Defining nutrients that support MT production will enable a more reliable identification of hazardous *S. chartarum* isolates. The new medium will also be instrumental in analyzing the biosynthetic pathways and regulatory mechanisms that control mycotoxin production in *S. chartarum.*

## INTRODUCTION

Stachybotrys chartarum (*Hypocreales*, *Ascomycota*) is one of the most frequently isolated species of the genus *Stachybotrys* (1). This filamentous fungus occurs ubiquitously in the environment and is commonly found in water-damaged buildings and construction materials (e.g., wallpaper and gypsum) as well as on dead plant material (e.g., straw and hay), culinary herbs, and marine sponges (2–7). *S. chartarum* is capable of producing a large variety of structurally diverse secondary metabolites, including trichothecenes, atranones, phenylspirodrimanes, and others (8). The species *S. chartarum* can be subdivided into (i) two distinct chemotypes based on their ability to produce either atranones (chemotype A) or macrocyclic trichothecenes (MTs; chemotype S) (9) or (ii) three genotypes A, S, and H according to the presence or absence of genes that are presumed to encode relevant enzymes for the biosynthesis of these mycotoxins (*atr*1‐14 and *sat*1‐21) (10).

*S. chartarum* strains have been implicated in several types of intoxications (11–17). In animals, especially horses, stachybotryotoxicosis can occur after oral ingestion or inhalation of mycotoxins from improperly stored moldy feed or, more rarely, after cutaneous contact (18–20). Humans, in particular infants, are primarily at risk in water‐damaged, mold-loaded buildings (14, 21, 22). *S. chartarum* can provoke symptoms related to the sick-building syndrome complex and is the suspected cause of pulmonary hemorrhage in infants after the uptake of airborne toxins (11, 15, 17). In addition, cases of stachybotryotoxicosis have been reported for farm workers who have handled *Stachybotrys*-contaminated straw or hay and in regions where hay or straw was used as bedding material (23, 24).

Trichothecenes structurally comprise four main groups: the trichothecenes types A, B, and C as well as D, which comprises the MTs (25–27). MTs represent the most toxic trichothecene group and include roridins, verrucarins, and satratoxins. *S. chartarum* genotype S strains are potent producers of roridin E (RE) and roridin L-2 (RL-2), satratoxins G, H, and F (SG, SH, and SF, respectively), and verrucarin J (VJ) (26, 28–30). MTs possess a marked cyto- and neurotoxic potential for mammals by blocking protein, DNA, and RNA biosynthesis (31–33) and induction of apoptosis (34, 35). However, MTs do not only possess a hazardous potential for humans and animals and are also reported to have a selective cytotoxic impact on certain human cancer cell lines, and Yang et al. emphasized the importance of SF (36). An inhibitory effect on tumor-related tyrosine kinases was described and attributed to RE, SG, and SH (7).

Phenylspirodrimanes (PSDs; e.g., stachybotrylactam [STLAC]) represent the largest and most abundant class of secondary metabolites produced by *Stachybotrys* species (37, 38). Despite this, their role in human and animal health is still unclear, but they are presumed to possess an immunosuppressive activity through inhibition of the complement system (39, 40).

Fungi use elaborate pathways that involve many different enzymes to produce mycotoxins and other secondary metabolites. The corresponding genes are commonly clustered, and their expression is highly variable and regulated by a variety of factors. To identify isolates that produce hazardous mycotoxins, it is of prime importance to use media that allow a robust and reliable production of these molecules. Factors known to influence mycotoxin biosynthesis are the time of growth, temperature, humidity, and nutrients (38, 41, 42). Regarding the impact of nutrients, the available nitrogen and carbon sources play a major role in these processes. This is not surprising considering the fact that nitrogen and carbon are essentially required for proteins, nucleic acids, and other cell substances. Their constant supply is therefore a prerequisite for fungal growth, including the production of secondary metabolites (43, 44).

The production of alternariol and alternariol monomethyl ether in Alternaria alternata (*Pleosporales*, *Ascomycota*) and of ochratoxin A (OTA) in Aspergillus species (*Eurotiales*, *Ascomycota*) clearly depends on the available nitrogen and carbon sources (45–47). For the latter, increased OTA production is triggered by lower levels of nitrogen and higher carbon concentrations (46). In Fusarium *proliferatum* (*Hypocreales*, *Ascomycota*), stress by nitrogen starvation induces the expression of genes for the biosynthesis of fumonisins (48), whereas high concentrations of nitrogen repress fumonisin production (49).

Despite their medical relevance, little is known about the factors that control the production of the mycotoxins in *S. chartarum*. Time of growth, humidity, and temperature have been investigated for their impact on growth and mycotoxin production in *S*. *chartarum* (38, 41, 50). Moreover, different building materials and certain commercially available media have been tested (51–54). Materials and media that are rich in cellulose and low in nitrogen as well as potato dextrose agar (PDA) were described to stimulate the production of mycotoxins (4, 54–56). Jarvis et al. (56) described that optimum mycotoxin production for the generation of higher quantities of satratoxins can be achieved with parboiled Ben’s Original rice as a solid culture medium. However, PDA and rice contain complex components, which makes it difficult to identify factors that influence mycotoxin production. Another drawback of PDA is that its complex components can vary, and this can have a dramatic impact on the amounts of mycotoxins that are produced (57). In other fungi, defined media have been established, such as Aspergillus minimal medium (AMM) (58), in which all ingredients are known and can be replaced or varied in concentration. For *S. chartarum*, such a medium so far is unknown, which complicates investigations of metabolic and biosynthetic processes.

The aim of this study was to investigate the impact of several nitrogen and carbon sources on mycelial growth and mycotoxin production of three genotype S strains of *S. chartarum*. AMM was identified as a suitable and well-defined nutrition medium, a finding that will facilitate a standardization of mycotoxin research in *S. chartarum* and will also be helpful in the analysis of the biosynthetic and metabolic pathways as well as the regulatory genes that control mycotoxin production in *S*. *chartarum*. Three nitrogen sources were tested in different concentrations and in combination with six carbon sources.

## RESULTS

The starting point of this study was the observation that the cultivation of *S. chartarum* in AMM results in strongly sporulating and extensively growing colonies comparable to PDA. This finding enabled us to analyze various carbon and nitrogen (N) sources for their impact on the toxin-forming capacity of *S. chartarum*. We included three *S. chartarum* strains in this study that are known for their effective production of macrocyclic trichothecenes (MTs) and stachybotrylactam (STLAC) (6, 9, 38, 54). Since our previously published data demonstrated that satratoxin production is tightly linked to sporulation (57), this work considered the size of the resulting colonies and their level of sporulation. The selected strains come from three substrates, namely, oats (ATCC 34916), building material from an apartment in Oakland (IBT 40293), and oregano (DSM 114129).

### Influence of various nitrogen sources on fungal growth and sporulation.

The influences of the nitrogen sources sodium nitrate (NaNO_3_), ammonium nitrate (NH_4_NO_3_), and ammonium chloride (NH_4_Cl) were analyzed at different concentrations that corresponded to 0, 1, 5, 10, 25, 50, 250, and 500 mg N/L. Nitrate (NO_3_^−^) and ammonium (NH_4_^+^) are the major inorganic nitrogen sources present in the environment and are assimilated by distinct mechanisms. We have therefore analyzed their impact either as sole nitrogen sources (NaNO_3_, NH_4_Cl) or as a combination of both sources (NH_4_NO_3_). All samples in these initial experiments contained 10 g/L glucose as the sole carbon source.

**FIG 1:**
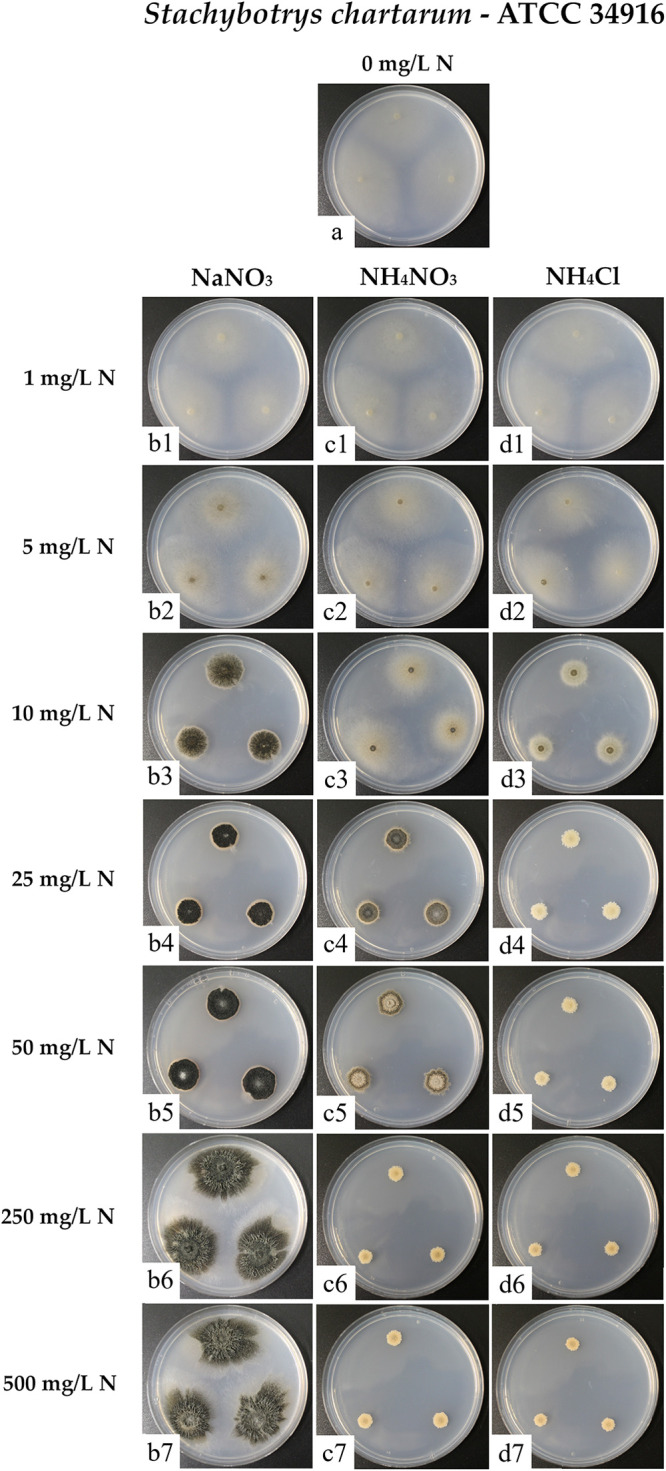
Colonies of *S. chartarum* genotype S strain ATCC 34916 were grown as three-point cultures on AMM containing glucose (10 g/L) as the sole carbon source. The different panels show cultures without nitrogen addition (a) or supplemented with NaNO_3_ (b1 to b7), NH_4_NO_3_ (c1 to c7), or NH_4_Cl (d1 to d7) at the following nitrogen concentrations: 1 mg/L (b1/c1/d1), 5 mg/L (b2/c2/d2), 10 mg/L (b3/c3/d3), 25 mg/L (b4/c4/d4), 50 mg/L (b5/c5/d5), 250 mg/L (b6/c6/d6), and 500 mg/L (b7/c7/d7). Each image is representative of three parallel cultures per condition.

The visually evaluable parameters were similar for all three strains; representative results obtained with strain ATCC 34916 are shown in Fig. 1. The colonies formed by IBT 40293 and DSM 114129 are presented in Fig. S1 in the supplemental material, and data on the colony areas of all three strains are summarized in Table S1.

The different nitrogen sources and their concentrations (normalized as mg N/L) had a striking impact on the size, morphology, and sporulation of the colonies. Large but flat and hardly sporulating colonies were found on plates containing small amounts of nitrogen (1 and 5 mg N/L) regardless of the chemical nature of the nitrogen source. A very similar growth phenotype was observed for plates without a nitrogen source, which suggests that a scarcity of nitrogen triggered an extensive spreading with hyphae that explored the environment for substrates richer in nitrogen. A comparison of the plates containing ≥10 mg N/L identified NaNO_3_ as the preferable nitrogen source. Smaller but dense, very dark, and therefore strongly sporulating colonies were found on plates containing 25 and 50 mg N/L. At higher concentrations (250 and 500 mg N/L), the colonies grown on NaNO_3_ were also substantially larger than the colonies grown on the lower nitrogen concentrations and resulted in large, dense, and well-sporulating colonies. NH_4_Cl was surprisingly a much poorer nitrogen source than NaNO_3_. Compared to the lower concentrations, 10 mg N/L NH_4_Cl triggered the formation of smaller colonies with a small, dark zone in the center that indicates the onset of sporulation. Remarkably, this positive trend was reversed on plates containing higher NH_4_Cl concentrations (25 to 500 mg N/L), where small, nonsporulating colonies occurred. The colonies on plates containing NH_4_NO_3_ were, at most concentrations, similar to those on plates supplemented with NH_4_Cl. The only exception was at 25 mg N/L, when colonies on plates with NH_4_NO_3_ had a more intermediate phenotype. These data indicate that sporulation was effectively prevented if NH_4_^+^ was present in concentrations corresponding to 25 mg N/L or more, regardless of whether NO_3_^−^ was present or not. In conclusion, these results demonstrate that NaNO_3_ is a superior nitrogen source for *S. chartarum* and that NH_4_^+^ was able to override the positive impact of NO_3_^−^ on growth and sporulation but only if it was present at concentrations above 25 mg N/L.

### Influence of different carbon sources on fungal growth and sporulation.

**FIG 2:**
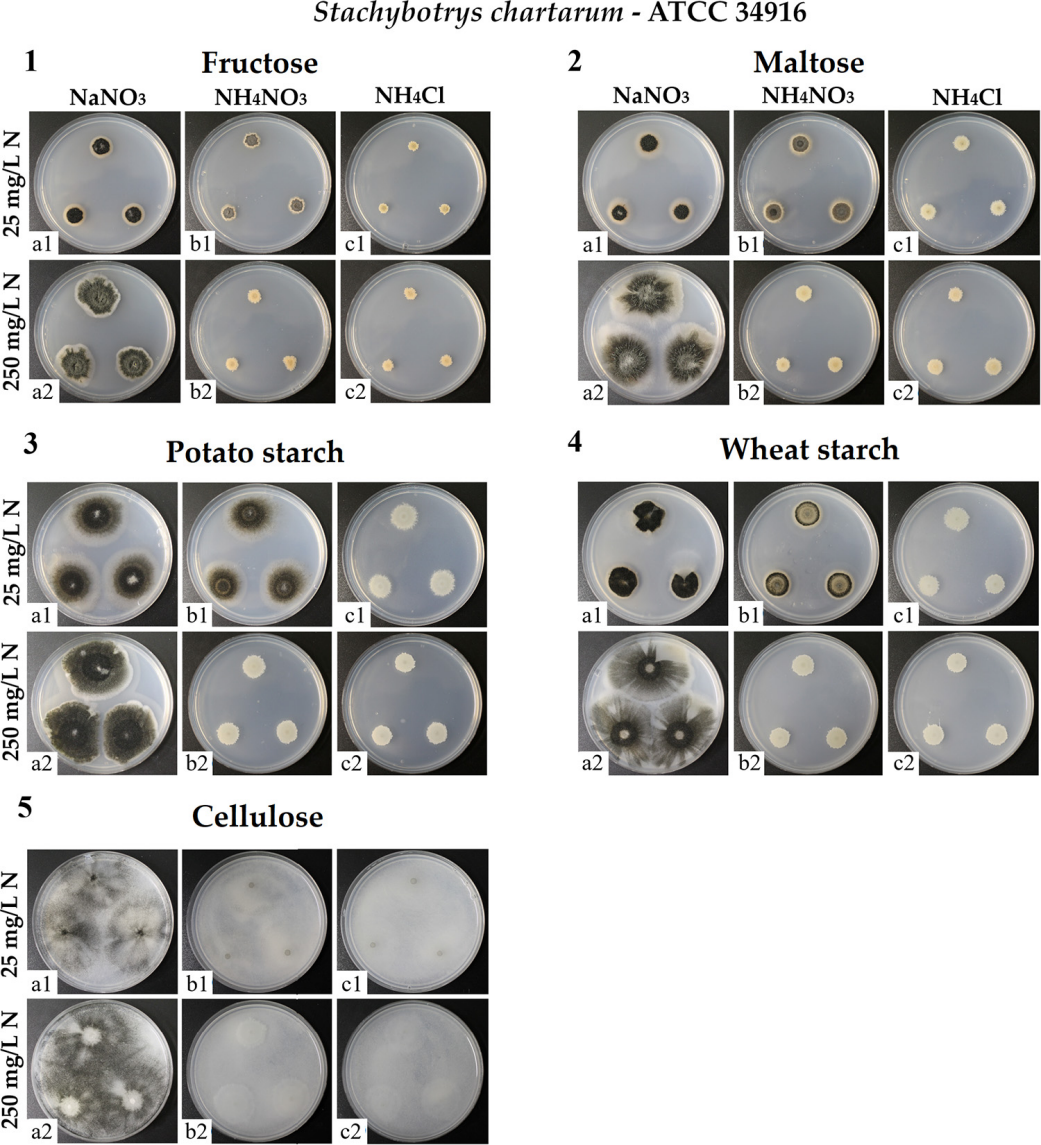
Colonies of *S. chartarum* genotype S strain ATCC 34916 were grown as three-point cultures on AMM containing fructose (1), maltose (2), potato starch (3), wheat starch (4), or cellulose (5) as the sole carbon source (with concentrations that were normalized to 4 g C/L) and combined with the nitrogen sources NaNO_3_ (1a1 to 5a1 and 1a2 to 5a2), NH_4_NO_3_ (1b1 to 5b1 and 1b2 to 5b2), or NH_4_Cl (1c1 to 5c1 and 1c2 to 5c2), each at two concentrations (25 and 250 mg N/L). Each image is representative of three parallel cultures per condition.

In the next step, we investigated the impact of different carbon sources, namely, glucose, fructose, maltose, potato starch, wheat starch, and cellulose in combination with nitrogen sources NaNO_3_, NH_4_NO_3_, and NH_4_Cl. As carbon sources, we selected mono-, di-, and polysaccharides that are commonly used in culture media or that were described to enable efficient growth of *S. chartarum*. The aim was to determine whether the chemical structure of the carbon source has an influence on growth and sporulation, as the assimilation of polysaccharides is an elaborate process that requires different enzymatic systems to degrade a polymer and to take up monomeric sugars. Because the results obtained in the analysis of nitrogen impact and described above were similar for all three strains tested, the first set of these experiments was performed only with strain ATCC 34916 (Fig. 2).

The most significant observation for all carbon sources tested was the consistently positive effect of NaNO_3_ on mycelial growth and sporulation and the comparatively negative effect of NH_4_NO_3_ and NH_4_Cl, results that resemble those obtained with glucose as the sole carbon source. It was noticeable that sporulation was completely prevented by NH_4_Cl concentrations of ≥25 mg N/L and an NH_4_NO_3_ concentration of 250 mg N/L, which underlines the dominant-negative effect of NH_4_^+^ when combined with NO_3_^−^. With respect to the size of the colonies, those formed with NH_4_NO_3_ and NH_4_Cl at 250 mg N/L were, on average, 88.1 ± 7.5% smaller than those formed with NaNO_3_ (*P* < 0.05) (Table S2). The only carbon source that led to a distinct colony phenotype was cellulose. These colonies spread extensively (regardless of the nitrogen source and its concentration), and on plates with NaNO_3_ (25 + 250 mg N/L) and NH_4_NO_3_ (25 mg N/L), the mycelium covered the whole surface of the plates.

One aspect was common to all cultures: only NaNO_3_ led to consistent and strong sporulation (Fig. 2). Thus, the inhibitory effect of NH_4_^+^ and the beneficial effect of NO_3_^−^ ions were equally pronounced for all carbon sources tested.

In further experiments, we compared the influence of the different carbon sources on all three genotype S strains. In this experiment, we used NaNO_3_ as the sole nitrogen source at a concentration of 250 mg N/L, a condition that enabled strong growth and sporulation in the initial experiments with ATCC 34916.

**FIG 3:**
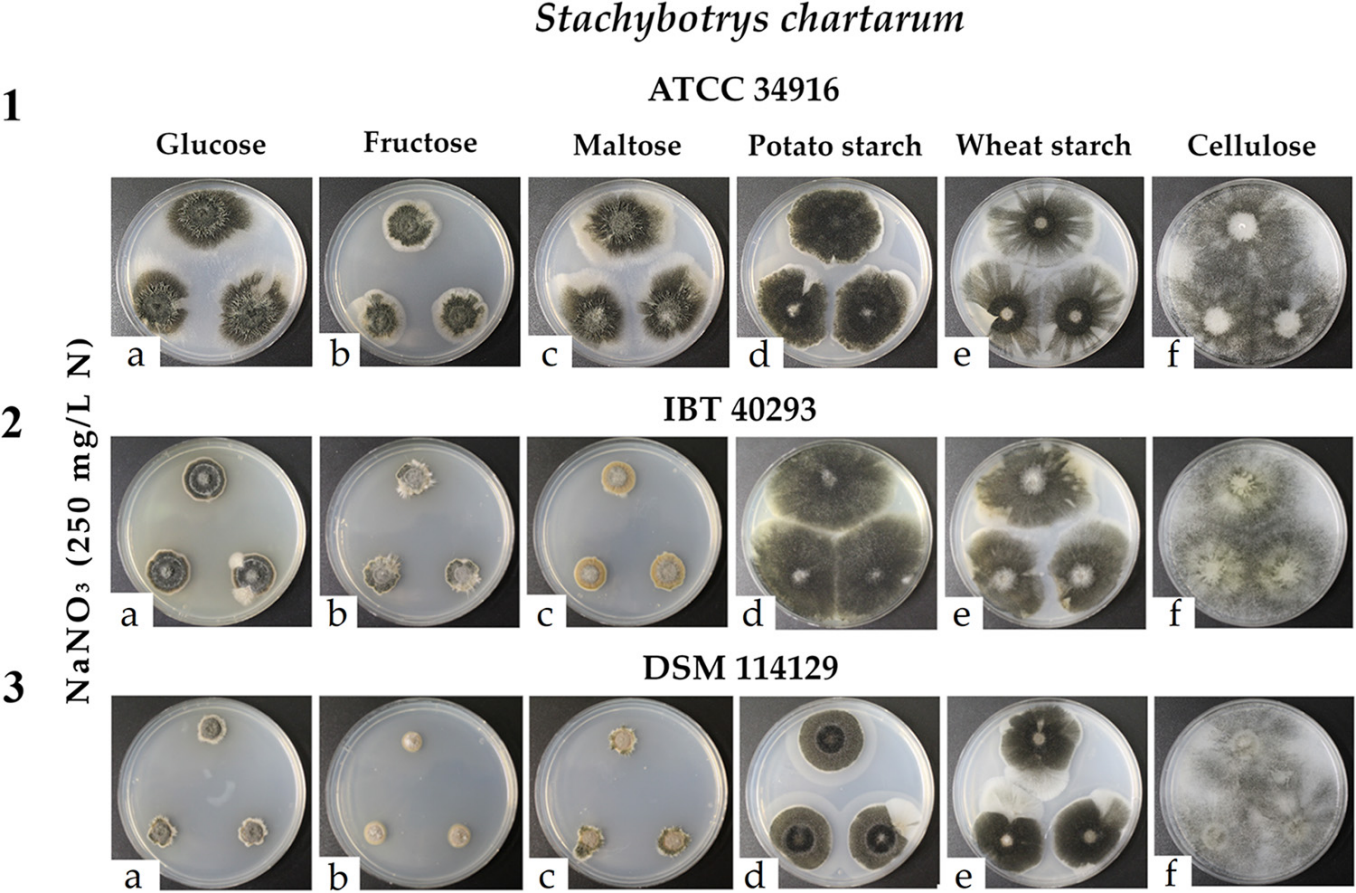
Colonies of *S. chartarum* genotype S strains ATCC 34916 (1), IBT 40293 (2), and DSM 114129 (3) were grown as three-point cultures on AMM containing glucose (1a to 3a), fructose (1b to 3b), maltose (1c to 3c), potato starch (1d to 3d), wheat starch (1e to 3e), or cellulose (1f to 3f) as the carbon source (with concentrations that were normalized to 4 g C/L) and NaNO_3_ as the nitrogen source (250 mg N/L). Each image is representative of three parallel cultures per condition.

Comparison of the different cultures revealed that not only the nitrogen source but also the carbon source (each normalized to 4 g C/L) had a striking impact on the size, morphology, and sporulation of the resulting colonies (Fig. 3; Table S2). Cellulose was again the only carbon source that led to a distinct phenotype. The fungal colonies spread extensively, and their flat and weakly sporulating mycelium covered the complete available surface of the plates (60.8 ± 0.0 cm^2^), indicating that the size of these colonies was clearly limited by the dimension of the plate. A very similar growth phenotype had been observed for plates with a low supply of nitrogen, suggesting that both a scarcity of nitrogen and carbon causes a hunger phenotype that was characterized by extensive hyphal spreading and colonies of low density.

Potato and wheat starch, the other two complex carbon sources, had a different effect than cellulose. Colonies grown on starch-containing medium were large, and their dark appearance indicated an extensive level of sporulation.

Differences between the three strains analyzed became evident on plates containing the mono- and disaccharides glucose, fructose, and maltose. Strain ATCC 34916 grew and sporulated best on these plates, strain IBT 40293 showed intermediate growth, and strain DSM 114129 showed the weakest growth. For the two latter strains, sporulation was best on glucose, and the growth of DSM 114129 was particular impaired on fructose and maltose, resulting in small colonies with very little visible sporulation. Thus, compared with starch, the tested mono- and disaccharides were clearly inferior carbon sources, and this was particular obvious for IBT 40293 and DSM 114129.

### Production of macrocyclic trichothecenes.

To evaluate the effect of different nitrogen and carbon sources on MT production, toxins were extracted from selected cultures and purified and analyzed by liquid chromatography-tandem mass spectrometry (LC-MS/MS). We considered three ways to normalize the data: amount of mycotoxin per plate, amount of mycotoxin per cm^2^ of colony area, or amount of mycotoxin per gram of mycelium. The latter appeared to be the best option. On the one hand, it turned out that it was technically impossible to harvest all fungal material from the plates. On the other hand, we realized that mycotoxins diffuse into the medium and that the analysis of pure fungal material would therefore not reflect the total amount of mycotoxins produced. The presentation as amount of mycotoxin per plate had the limitation that different culture conditions led to colonies of variable size, which had a strong impact on the calculated values. To avoid this limitation, we also calculated the MT concentrations per cm^2^ of colony area.

### (i) Influence of various nitrogen sources on the production of macrocyclic trichothecenes.

The impact of NaNO_3_, NH_4_NO_3_, and NH_4_Cl on the production of MTs was investigated using cultures of the three genotype S strains grown on plates containing 1, 25, or 250 mg N/L and 10 g glucose/L (Fig. 1; Fig. S1). After acetonitrile/water (ACN/H_2_O) extraction (84/16 [vol/vol]), the purified toxin extracts were analyzed by LC-MS/MS for their content of roridin E (RE), roridin L-2 (RL-2), verrucarin J (VJ), verrucarin A (VA), satratoxin G (SG), satratoxin H (SH), and satratoxin F (SF).

As shown in Table S3, an increase in NaNO_3_ concentrations had a positive effect on the total amount of MTs per agar plate (ng/agar plate). This effect was observed for all three strains tested. In the case of ATCC 34916, the cultures that were grown with a nitrogen concentration of 25 mg N/L produced approximately seven times more MTs than those grown with 1 mg N/L. In turn, the cultures grown on medium containing 250 mg N/L produced approximately 1.8 times more MTs than those grown with 25 mg N/L.

For NH_4_NO_3_, increasing the concentration from 1 mg N/L to 25 mg N/L also resulted in increasing MT concentrations per plate for all three strains (e.g., approximately 4-fold for ATCC 34916), but a further increase to 250 mg N/L abrogated MT production (values below the limit of detection [LOD]). When NH_4_Cl was used as the sole nitrogen source, MTs were only detectable at low concentrations and only in cultures grown with 1 mg N/L.

Strikingly, the large colonies found on plates containing 1 mg N/L produced only small amounts of MTs per plate, demonstrating that MT production was not proportional to the colony size but rather to the level of sporulation.

Taken together, for all three strains, NaNO_3_ was the superior nitrogen source to trigger MT production, and the highest toxin concentrations per plate were produced when NaNO_3_ was added at the highest concentration tested. Growth on NH_4_Cl-containing plates led to very low or even undetectable levels of MT production, in particular in cultures containing 25 and 250 mg N/L. For NH_4_NO_3_, concentrations of up to 25 mg N/L stimulated MT production, but it was strongly repressed and below the LOD at 250 mg N/L. In conclusion, the impact of the three different nitrogen sources on MT production was very similar to that on growth and especially on sporulation, or, in other words, large and in particular strongly sporulating colonies formed the largest amounts of MTs per agar plate.

**FIG 4:**
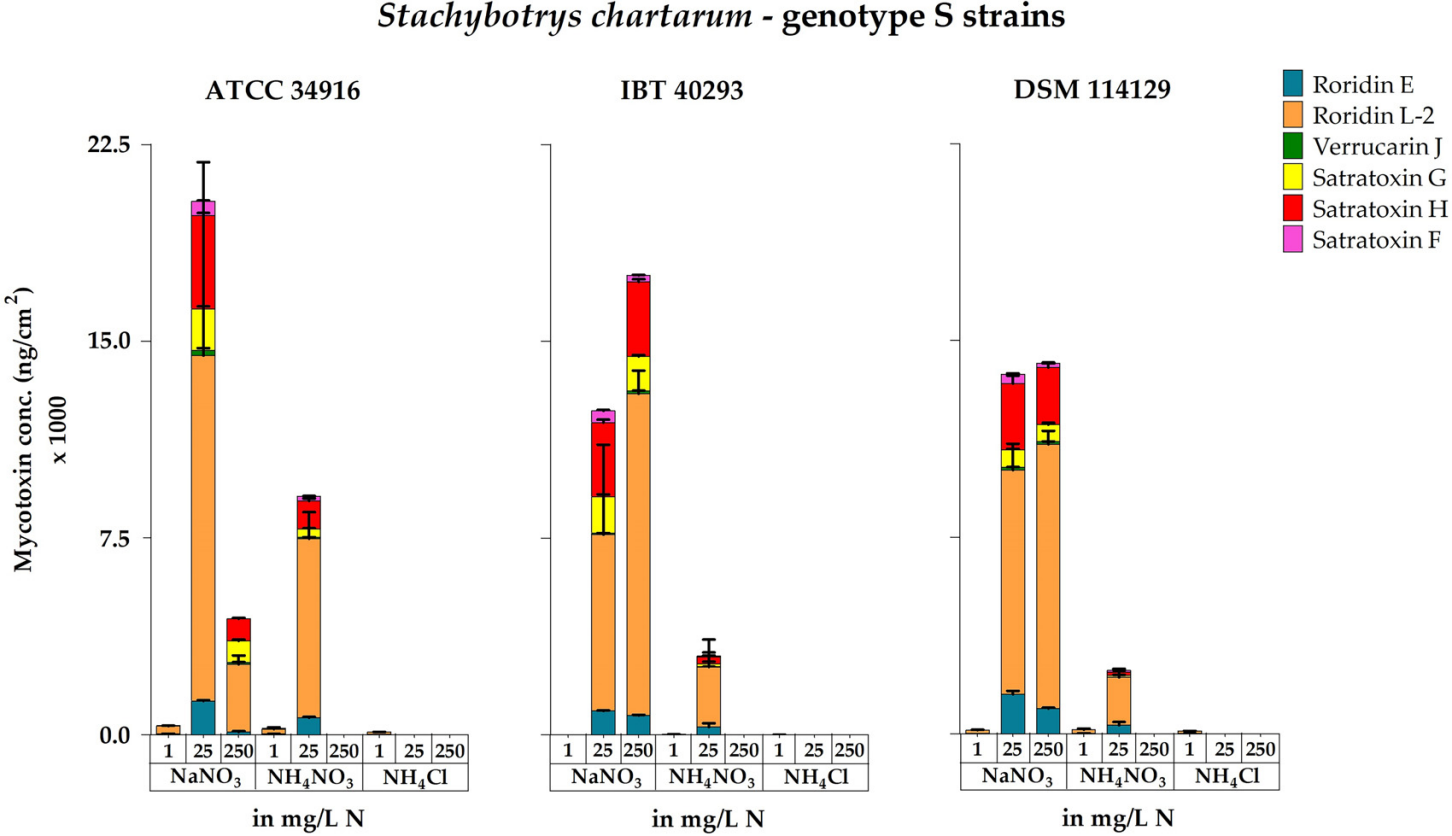
Accumulated concentrations of the macrocyclic trichothecenes roridin E, roridin L-2, verrucarin J, satratoxin G, satratoxin H, and satratoxin F measured for cultures of *S. chartarum* genotype S strains ATCC 34916, IBT 40293, and DSM 114129 grown on AMM supplemented with NaNO_3_, NH_4_NO_3_, or NH_4_Cl as the nitrogen source at concentrations of 1, 25, or 250 mg N/L. In this figure, the concentrations are normalized to the area of the respective colonies (ng/cm^2^). For representative images of these corresponding cultures, compare Fig. 1 and Fig. S1 in the supplemental material.

When calculated as MT production per cm^2^ of colony area, the results again revealed similarities between the three strains: NaNO_3_ was the superior nitrogen source and NH_4_Cl was the inferior nitrogen source to trigger MT production. NH_4_NO_3_ stimulated toxin production at low concentrations but also abrogated toxin production at high concentrations (1 and 25 versus 250 mg N/L) (Fig. 4).

The large colonies of ATCC 34916 on plates containing 250 mg N/L produced significantly lower levels of MTs per cm^2^ of colony area than the smaller colonies grown with 25 mg N/L (*P* < 0.05). This is different to the results calculated per plate, but in both experiments, the amounts of MTs correlated well with the respective level of sporulation.

**FIG 5:**
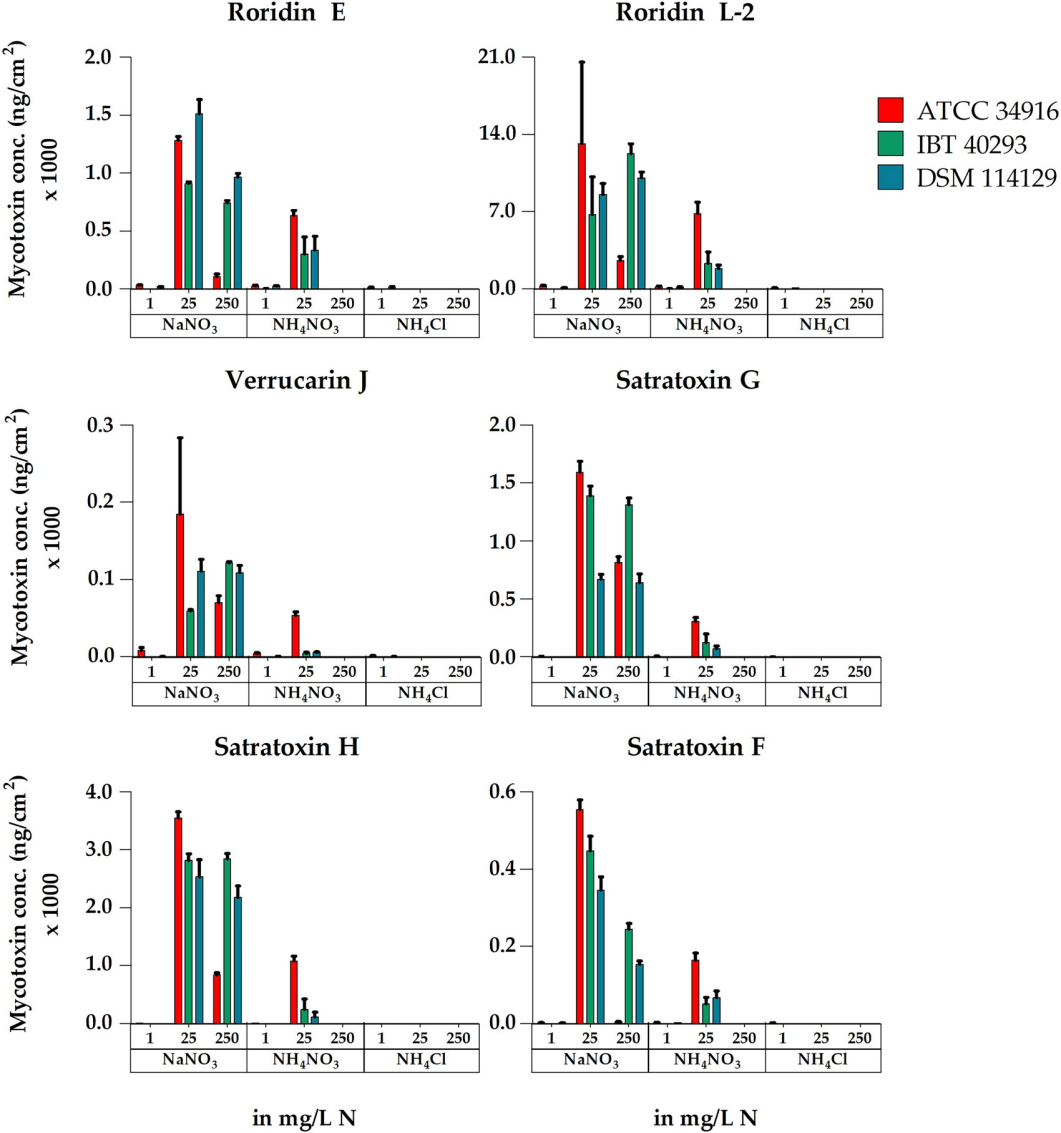
Concentrations of the macrocyclic trichothecenes roridin E, roridin L-2, verrucarin J, satratoxin G, satratoxin H, and satratoxin F measured for cultures of *S. chartarum* genotype S strains ATCC 34916 (red columns), IBT 40293 (green columns), and DSM 114129 (blue columns) grown on AMM supplemented with NaNO_3_, NH_4_NO_3_, or NH_4_Cl at concentrations of 1, 25, or 250 mg N/L. The concentration values are normalized to the area of the respective colonies (ng/cm^2^).

In the following, we also considered every single MT that was investigated. The LC-MS/MS analysis revealed that the overall pattern of MTs was comparable for the three strains and differed only in their individual concentrations, a finding that is in line with previous studies (54). Each strain produced RL-2, RE, VJ, SG, SH, and SF. In all samples, RL-2 was the most abundant MT, and its highest concentrations were obtained with NaNO_3_ (Table S3). RE, SG, and SH were produced in lower concentrations, whereby more SH was produced than SG (79%). We also screened the samples for VJ and SF, but the respective values were the lowest of all MTs analyzed. VA was not detectable in any of the samples, which is in line with previous studies (54, 59). The data for the production of individual MTs largely mirrored all trends described above for accumulated MT production, a positive impact of NaNO_3_ and a negative, or even inhibitory, effect of NH_4_NO_3_ and NH_4_Cl, especially at higher concentrations (Fig. 5).

### (ii) Influence of different carbon sources on the production of macrocyclic trichothecenes.

We also analyzed the potential impact of different carbon sources in combination with NaNO_3_, NH_4_NO_3_, or NH_4_Cl on MT production. If the results obtained with strain ATCC 34916 are calculated as accumulated MT production per plate (Table S4), all cultures reproduced the above-described trends observed with glucose; NaNO_3_ stimulated and NH_4_^+^ repressed MT production, especially at higher concentrations. Again, large and strongly sporulating colonies formed the largest amounts of MTs. Because the positive effect of NaNO_3_ and the negative effect of NH_4_^+^ on MT production were equally pronounced for all carbon sources tested, combinatorial effects could be excluded, a result that resembled that obtained for mycelial growth and sporulation. The MT concentrations in the individual cultures were nevertheless dependent on the available carbon source, reaching the highest levels with potato starch (369.7 ± 17.6 μg/agar plate) and the lowest levels with fructose (73.9 ± 12.1 μg/agar plate).

**FIG 6:**
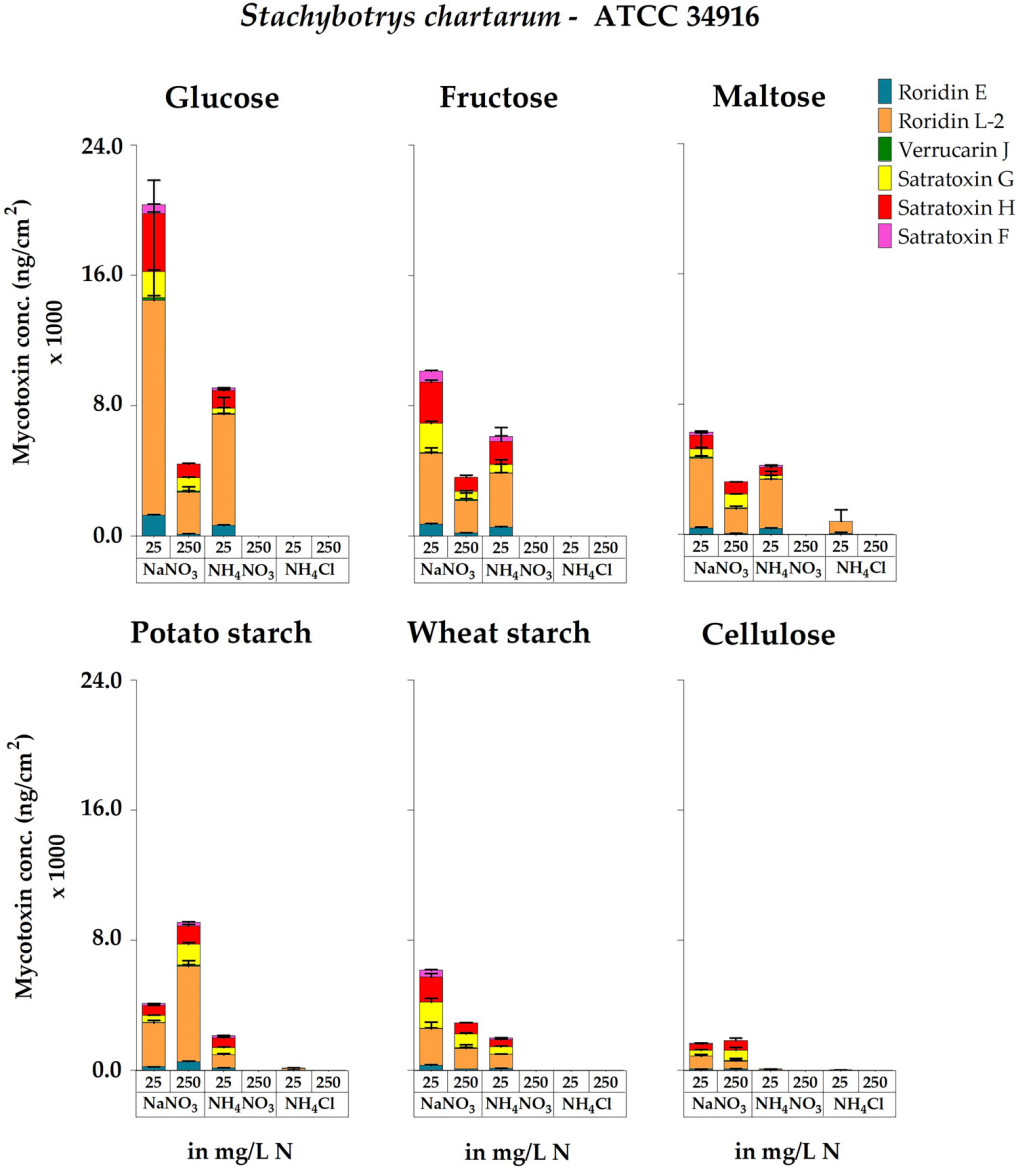
Accumulated concentrations of the macrocyclic trichothecenes roridin E, roridin L-2, verrucarin J, satratoxin G, satratoxin H, and satratoxin F measured for cultures of *S. chartarum* genotype S strain ATCC 34916 grown on AMM containing glucose, fructose, maltose, potato starch, wheat starch, or cellulose as the sole carbon source (with concentrations that were normalized to 4 g C/L) and combined with the nitrogen sources NaNO_3_, NH_4_NO_3_, or NH_4_Cl (either 25 or 250 mg N/L). The MT concentrations are normalized to the area of the respective colonies (ng/cm^2^). For representative images of these corresponding cultures, compare Fig. 1 and 2.

If MTs were calculated in ng per cm^2^ of colony area (Fig. 6), the data revealed a correlation between sporulation and MT production. Larger colonies grown on medium containing fructose, maltose, or wheat starch and a higher NaNO_3_ concentration (250 mg N/L) resulted in a high yield of MTs per plate, but the toxin amounts per cm^2^ were higher for the smaller, darker, and more densely sporulating colonies grown with lower concentrations of NaNO_3_ (25 mg N/L).

The highest levels of MTs per cm^2^ of colony area were reached with glucose (20.3 ± 7.4 μg/cm^2^) and fructose (10.1 ± 0.2 μg/cm^2^) in combination with the lower NaNO_3_ concentration and potato starch (9.1 ± 0.5 μg/cm^2^) combined with the higher NaNO_3_ concentration. The lowest levels of MT production were found for colonies grown with cellulose (1.7 ± 0.2 μg/cm^2^).

At the level of individual MT characterization, again NaNO_3_ was superior to NH_4_^+^ as the nitrogen source, particularly when present at higher concentrations (Table S4). Strain ATCC 34916 produced RL-2, RE, VJ, SG, SH, and SF but no VA. RL-2 was the most abundant, and VJ and SF were the MTs produced at the lowest amounts.

Effects of the different carbon sources on all three genotype S strains were compared using the cultures shown in Fig. 3. Here, NaNO_3_ was used as the sole nitrogen source at a concentration of 250 mg N/L, a condition that led to strong sporulation in the initial experiments with ATCC 34916.

Comparison of the total amounts of MTs produced per plate (Table S4) demonstrated that the cultures grown with potato starch produced the highest levels of MTs. The highest concentration was measured for IBT 40293, which was significantly higher (*P* < 0.05; 632.6 ± 32.9 μg/agar plate) than those produced by ATCC 34916 and DSM 114129. The colonies grown on plates containing wheat starch or cellulose produced generally lower levels of MTs.

The MT concentrations measured for colonies grown on plates containing the mono- and disaccharides glucose, fructose, and maltose revealed differences between the three strains. ATCC 34916 produced higher levels of MTs per plate, strain IBT 40293 showed an intermediate phenotype, and strain DSM 114129 showed the weakest level of MT production. The two latter strains formed substantial MT levels only on glucose. The lowest levels of MT production for ATCC 34916 and DSM 114129 were reached with fructose (73.9 ± 12.1 and 3.8 ± 0.4 μg/agar plate), while the lowest levels of MT production for IBT 40293 were reached with maltose (18.2 ± 9.5 μg/agar plate).

The tested mono- and disaccharides were clearly the inferior carbon sources and stimulated lower MT production than starch and cellulose, which was particularly obvious for IBT 40293 and DSM 114129.

**FIG 7:**
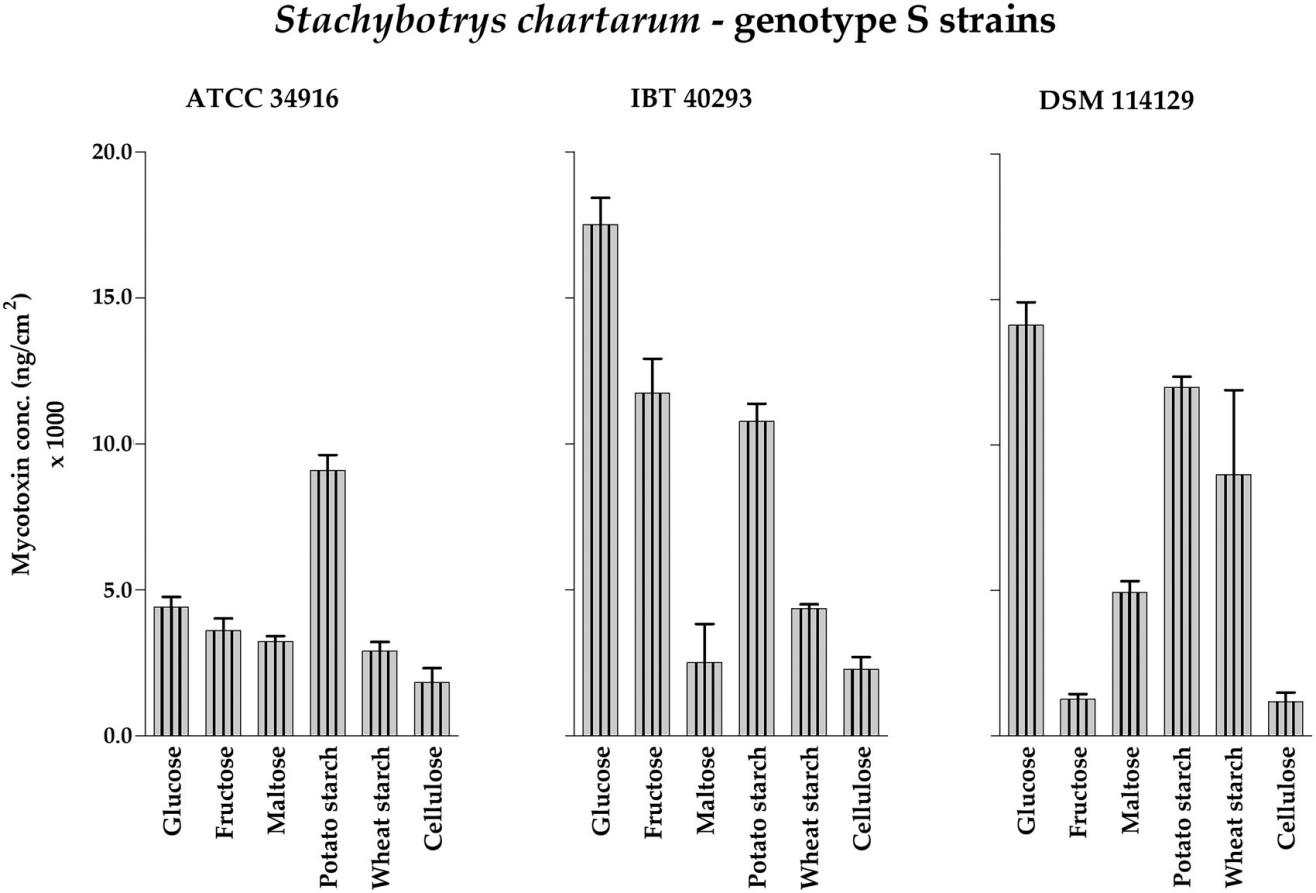
Accumulated concentrations of the macrocyclic trichothecenes roridin E, roridin L-2, verrucarin J, satratoxin G, satratoxin H, and satratoxin F measured for cultures of *S. chartarum* genotype S strains ATCC 34916, IBT 40293, and DSM 114129 grown on AMM containing glucose, fructose, maltose, potato starch, wheat starch, or cellulose as the carbon source (with concentrations that were normalized to 4 g C/L) and NaNO_3_ as the nitrogen source (with 250 mg N/L). The data are normalized to the area of the respective colonies (ng/cm^2^). For representative images of these corresponding cultures, compare with Fig. 3.

MT production calculated as total amount of MTs per cm^2^ of colony area (Fig. 7) revealed some differences between the six carbon sources: colonies grown on potato starch produced relatively high amounts of MTs (ATCC 34916: 9.1 ± 0.5 μg/cm^2^; IBT 40293: 10.8 ± 0.6 μg/cm^2^; DSM 114129: 12.0 ± 0.4 μg/cm^2^), and colonies grown on cellulose produced comparatively lower amounts (ATCC 34916: 1.8 ± 0.5 μg/cm^2^; IBT 40293: 2.3 ± 0.4 μg/cm^2^; DSM 114129: 1.2 ± 0.3 μg/cm^2^). These results resembled those obtained for colony growth and sporulation, as all three strains formed very dark and densely sporulating colonies when grown on potato starch but flat, weakly sporulating and flimsy colonies on cellulose.

In conclusion, considering the data for growth, sporulation, and MT production (calculated as either ng of MTs per plate or ng of MTs per cm^2^), potato starch was clearly the superior and most reliable carbon source for all three strains tested. Growth on potato starch resulted in large colonies with dense mycelium and high levels of sporulation and MT production.

### Production of stachybotrylactam.

To evaluate the effect of the different nitrogen and carbon sources on STLAC production, the same cultures already analyzed for MT production were also screened for STLAC production.

### (i) Influence of various nitrogen sources on the production of stachybotrylactam.

The impact of NaNO_3_, NH_4_NO_3_, and NH_4_Cl on the production of STLAC was investigated using cultures of the three genotype S strains grown on plates containing 1, 25, or 250 mg N/L and 10 g glucose/L (Fig. 1; Fig. S1). After toxin extraction, the purified samples were analyzed by LC-MS/MS, and their STLAC content was determined.

**FIG 8:**
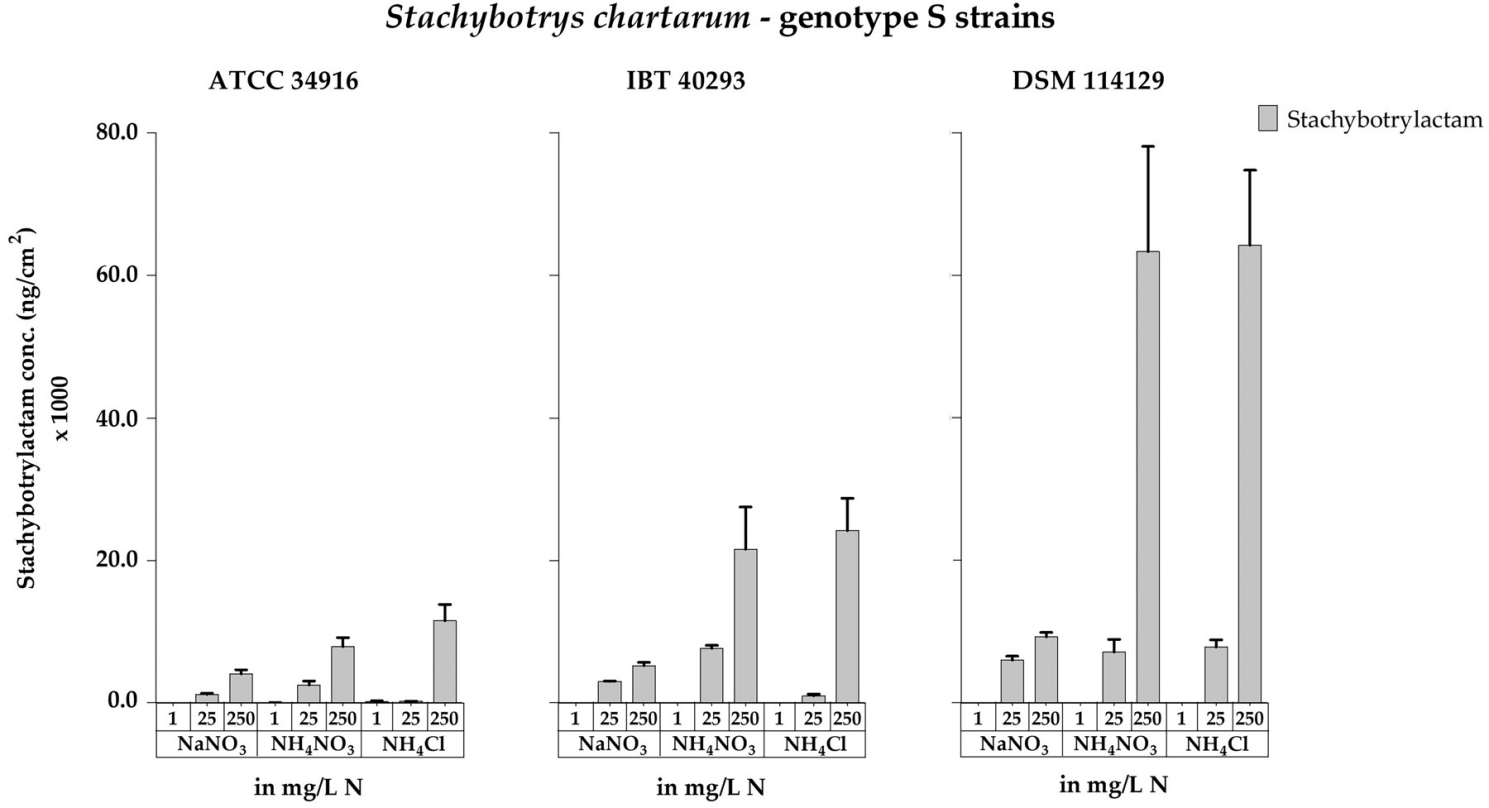
Stachybotrylactam concentrations measured for cultures of *S. chartarum* genotype S strains ATCC 34916, IBT 40293, and DSM 114129 grown on AMM supplemented with NaNO_3_, NH_4_NO_3_, or NH_4_Cl as the nitrogen source at concentrations of 1, 25, or 250 mg N/L. The data are normalized to the area of the respective colonies (ng/cm^2^). For representative images of these corresponding cultures, compare Fig. 1 and Fig. S1 in the supplemental material.

As shown in Fig. 8 and Table S5, increasing NaNO_3_ concentrations had a positive effect on STLAC production in all three strains, but NaNO_3_ was a comparatively inferior nitrogen source to trigger STLAC production, and significantly smaller amounts of STLAC (*P* < 0.05) were produced than observed when NH_4_NO_3_ and NH_4_Cl were the nitrogen sources, a pattern that was clearly different than that obtained for MT production (Fig. 4). The impacts of NH_4_NO_3_ and NH_4_Cl were very similar, and both nitrogen sources supported a strong production of STLAC. DSM 114129 was the best STLAC producer, while ATCC 34916 was the weakest. We observed no positive correlation between sporulation and STLAC production, since nonsporulating colonies also produced large amounts of STLAC.

### (ii) Influence of different carbon sources on the production of stachybotrylactam.

To determine a potential impact of different carbon sources on STLAC production, we also tested the cultures of strain ATCC 34916 shown in Fig. 2.

**FIG 9:**
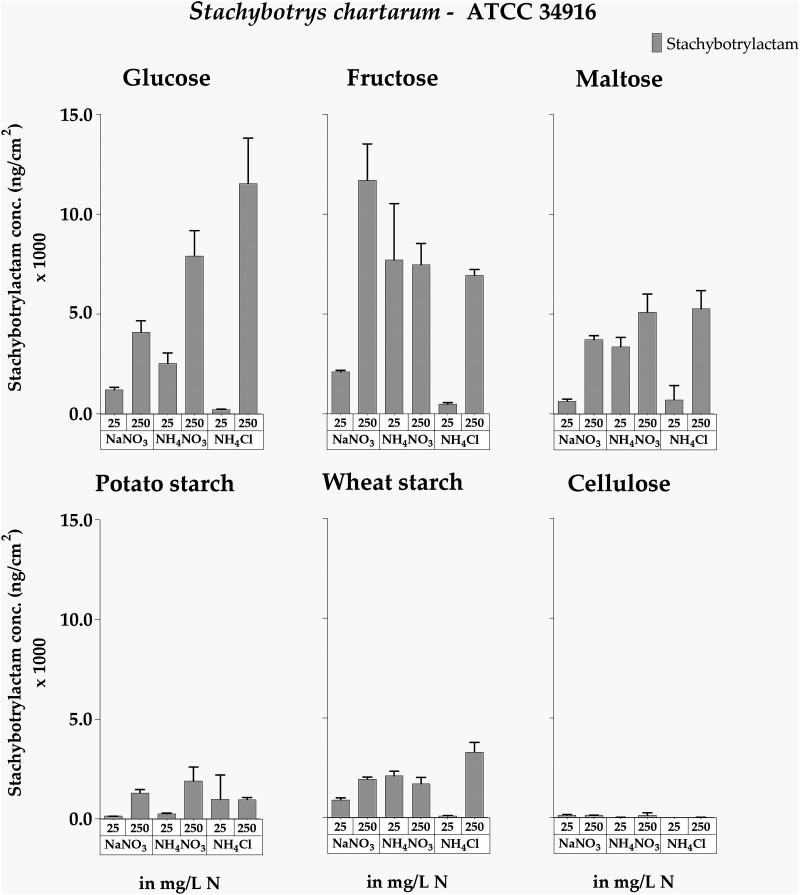
Stachybotrylactam concentrations measured for cultures of *S. chartarum* genotype S strain ATCC 34916 grown on AMM containing glucose, fructose, maltose, potato starch, wheat starch, or cellulose as the sole carbon source (with concentrations that were normalized to 4 g C/L) and combined with the nitrogen sources NaNO_3_, NH_4_NO_3_, or NH_4_Cl (either 25 or 250 mg N/L). The data are normalized to the area of the respective colonies (ng/cm^2^). For representative images of these corresponding cultures, compare Fig. 1 and 2.

The observation that NaNO_3_ was the inferior nitrogen source for STLAC production compared to NH_4_NO_3_ and NH_4_Cl could not be confirmed in this data set, but for ATCC 34916, this effect was also not particularly pronounced on the glucose-containing plates. For ATCC 34916, the impact of all nitrogen sources was similar in that all these sources supported stronger STLAC production at higher concentrations (Fig. 9; Table S6).

Comparison of the six different carbon sources revealed that, overall, STLAC production was more affected by the carbon source than by the nitrogen source. The mono- and disaccharides led to better STLAC production than the two types of starch and cellulose, and no correlation was evident between STLAC production and colony size or the level of sporulation. Again, these patterns are contrary to those observed for MT production.

In further experiments, we compared the influence of the different carbon sources on all three genotype S strains. In this experiment, NaNO_3_ was used as the sole nitrogen source at a concentration of 250 mg N/L. The corresponding cultures are shown in Fig. 3.

**FIG 10:**
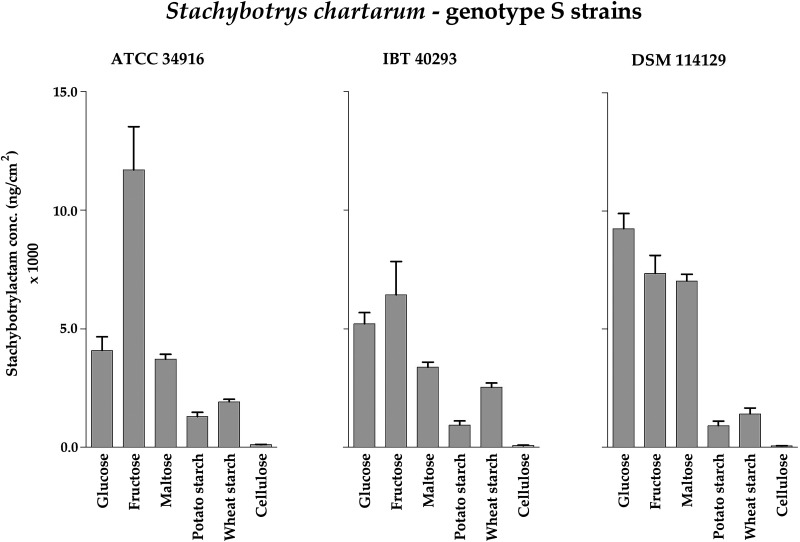
Stachybotrylactam concentrations measured for cultures of *S. chartarum* genotype S strains ATCC 34916, IBT 40293, and DSM 114129 grown on AMM containing glucose, fructose, maltose, potato starch, wheat starch, or cellulose as the carbon source (with concentrations that were normalized to 4 g C/L) and NaNO_3_ as the nitrogen source (with 250 mg N/L). The data are normalized to the area of the respective colonies (ng/cm^2^). For representative images of these corresponding cultures, compare Fig. 3.

For STLAC production in all three strains (Fig. 10; Table S6), the mono- and disaccharides yielded larger amounts than the two types of starch and in particular cellulose that nearly abolished STLAC production.

## DISCUSSION

Fungi are potent producers of a wide array of extra- and intracellular compounds, including secondary metabolites and other molecules. While some of these molecules are essential for fungal growth, many others are not directly required, and, in many cases, their benefit for the producing organism remains elusive (60). *Stachybotrys* species produce a broad spectrum of secondary metabolites, and, to date, more than 200 different compounds have been discovered, including macrocyclic trichothecenes (MTs) and phenylspirodrimanes (PSDs) (8). Most of these metabolites have been linked to either hazardous or beneficial health effects in humans and animals (30).

Despite their medical relevance, there is a substantial lack of knowledge about the impact of nutrients on mycotoxin production in *S. chartarum*. Several studies analyzed the effects of complex media and substrates on growth and toxin production (51, 53, 54), but this approach is not suitable to define the impact of single nutrients. In various fungi, it was shown that hyphal growth, mycotoxin profiles, and concentrations are significantly influenced by the available nutrients, especially the nitrogen and carbon sources (45–47). The strong effect of these compounds is not surprising considering the fact that nitrogen and carbon are essentially required for proteins, nucleic acids, and other cell substances, and their constant supply is a prerequisite for growth.

The observation that *S. chartarum* grows well in Aspergillus minimal medium (AMM) was the starting point of this study. It allowed us to analyze the impact of defined nitrogen and carbon sources on growth, sporulation, and mycotoxin production of *S. chartarum*. We studied three genotype S strains of the species *S. chartarum*. The selected strains originated from three substrates, namely, oats (ATCC 34916), building material from an apartment in Oakland (IBT 40293), and oregano (DSM 114129). Despite their different origins, the overall effect of nutrients on mycelial growth and mycotoxin production was well comparable for the three strains.

According to our data, the impact of the different nitrogen and carbon sources tested on growth and especially sporulation was very similar to that on MT production, or, in other words, large and strongly sporulating colonies formed the highest concentrations of MTs, and less or nonsporulating colonies formed lower or even undetectable amounts of MTs. Mycotoxins of several fungi are known to be associated with sporulation and are secreted by growing colonies (61), and a link between secondary metabolism and sporulation is a common feature in many filamentous fungi (62). Previously, similar results were obtained with *S. chartarum* (57), and, therefore, our observations are in good agreement with published data.

Due to different pathways that are engaged in nitrogen catabolism, many fungi are competent in using a large variety of nitrogen sources (63). NH_4_^+^ and NO_3_^−^ play a major role in various biochemical processes; they differ in the oxidation state of nitrogen and are suitable nitrogen sources for many fungi. In this study, we compared the impact of three inorganic nitrogen sources, namely, NaNO_3_, NH_4_NO_3_, and NH_4_Cl. The resulting colonies grown on AMM agar differed and revealed that each nitrogen source had a distinct impact.

NaNO_3_ was shown to be the superior nitrogen source for all three strains and allowed substantially better growth that resulted in dense and heavily sporulating mycelium with high MT production. Our data also imply that NaNO_3_ had to be present in concentrations corresponding to 10 mg N/L or higher to enable good growth of the cultures. NO_3_^−^ has to be reduced to the level of NH_4_^+^ before it can feed into metabolism, a process that requires genes that encode nitrate and nitrite reductases (64). Such genes are well defined in other fungi, for example, Neurospora crassa (*Sordariales*, *Ascomycota*) (65) and Aspergillus nidulans (*Eurotiales*, *Ascomycota*) (66), but are not yet identified in the genome of *S. chartarum*.

In contrast, NH_4_Cl was surprisingly a much poorer nitrogen source, and cultivation on NH_4_Cl-containing medium resulted in small, nonsporulating colonies with low or even undetectable MT production. A first hint that cultivation on NH_4_Cl-containing medium can lead to abrogation of sporulation of *Stachybotrys* species dates back to 1963 (67). Our data demonstrate that this impact is already evident at low concentrations (≥25 mg N/L). This negative impact of NH_4_^+^ is surprising since NH_4_^+^ ions can be directly used to produce glutamine from glutamate (68), whereas the assimilation of NO_3_^−^ is a more elaborate process. NH_4_^+^-mediated inhibition of sporulation has been reported for several fungi (69–71), but the underlying mechanism has not yet been disclosed.

The finding that NaNO_3_ enables better growth than NH_4_Cl is one of the key findings of this study. For different organisms, it is well known that due to the potential toxicity of NH_4_^+^, the sensing and uptake of this ion are carefully regulated by specific transporters and can be inhibited by regulatory proteins (72). Scarce growth with NH_4_^+^ salts was observed for different fungi. Brian, Curtis, and Hemming (73) suggested that certain types of organic acids are required during growth with NH_4_^+^ to provide keto acids that prevent an accumulation of toxic NH_4_^+^ ions in cells by stopping the uptake mechanisms until acceptor molecules are again available. According to an alternative explanation, the inhibitory effect of NH_4_^+^ results from a pH shift since the assimilation of NH_4_^+^ is always accompanied by a release of H^+^ cations and a concomitant acidification (74). The resulting weak assimilation can then be improved by addition of buffering organic acid salts (75). Hence, the influence of pH on growth, sporulation, and mycotoxin production should clearly be investigated in future studies. Another aspect that may be relevant in this context is that NO_3_^−^ could be used and could therefore be beneficial as an electron acceptor during nitrate respiration, a process that is found in many fungi (76). Alternative respiration pathways are used by certain fungi under oxygen limitation (77), although this condition is unlikely under the experimental settings of this study.

Interestingly, mycelial growth, sporulation, and MT production of colonies grown on NH_4_NO_3_ were more similar to those of colonies grown on NH_4_Cl, suggesting that the assimilation of NO_3_^−^ is suppressed, and the negative impact of NH_4_^+^ prevails. This regulatory system has not yet been described for *S. chartarum*, but it is known as nitrogen catabolite repression and has been found in various other fungi (78). When NH_4_^+^ is present, other genes that are involved in the acquisition and utilization of alternative nitrogen sources are repressed (68). This seems to have an adaptive significance in natural habitats, such as in soil, where NH_4_^+^ and NO_3_^−^ frequently occur together. Since more energy has to be expended for the assimilation of NO_3_^−^, it can be assumed that the latter is not preferentially used (75). Whether the mechanism of nitrogen catabolite repression applies for *S. chartarum* is an open question and clearly requires further studies.

Since for other fungi nitrogen concentration was described to have a major impact on the biosynthesis of mycotoxins (46, 48, 49), we analyzed this production at different nitrogen concentrations, namely, 0, 1, 5, 10, 25, 50, 250, and 500 mg N/L. For *S. chartarum*, there are hardly any data available on the impact of different nitrogen concentrations. Building materials have a low nitrogen content but have been described to provide suitable conditions for growth and toxin formation; however, the actual influence of nitrogen remained unclear (22). Ulrich et al. (54) also speculated on a possible effect of nitrogen concentration on the production of mycotoxins, since fewer mycotoxins were detected on nitrogen-rich complex media than on media with a lower nitrogen content. However, this study used complex, not well-defined media, which hampers any clear analysis of the impact of individual nutrients.

When *S. chartarum* was cultivated on higher concentrations of NH_4_^+^, MT production decreased to levels that were undetectable, whereas it increased clearly when cultivated on increasing concentrations of NO_3_^−^. The cultivation on small amounts of nitrogen (1 and 5 mg/L), regardless of their chemical nature, led to large but flat and hardly sporulating colonies. This suggests that a scarcity of nitrogen triggered an extensive spreading of hyphae that explored the environment for a substrate richer in nitrogen, which resulted in a small amount of fungal mass. Similar growth phenotypes have also been demonstrated for Candida albicans (*Saccharomycetales*, *Ascomycota*) and Saccharomyces cerevisiae (*Saccharomycetales*, *Ascomycota*). They are controlled by nitrogen transceptors, nitrogen transporters that act also as receptors and control downstream signaling pathways and enable survival in a nitrogen-depleted environment through the formation of pseudohyphae and filamentous growth toward new nitrogen sources (79, 80).

Since previous studies were not sufficient to describe the impact of different monosaccharides and polysaccharides on the growth and mycotoxin production of *Stachybotrys*, we tested the effects of different carbon sources, namely, glucose, fructose, maltose, potato starch, wheat starch, and cellulose. The different carbon substrates were combined with the three nitrogen sources NaNO_3_, NH_4_NO_3_, and NH_4_Cl. The two most significant observations of this study, the dominant inhibitory effect of NH_4_^+^ on mycelial growth, sporulation, and MT production and the beneficial effect of NO_3_^−^, were equally pronounced for all carbon sources tested. This implies that combinatorial effects could be largely excluded, and the effects of the nitrogen sources clearly prevail.

When the cultures grown on NaNO_3_-containing medium were compared, it became apparent that the carbon sources also had a striking impact on colony size, morphology, and sporulation and MT production. Colonies grown on starch-containing medium were strongly sporulating, dark, and composed of a dense mycelial network. The tested mono- and disaccharides were clearly inferior carbon sources compared with starch, and this was particularly obvious for IBT 40293 and DSM 114129. These observations are in good agreement with previous studies that proposed that potato dextrose agar is the optimal nutrition medium for growth and MT production (54, 81). Interestingly, strain ATCC 34916 grew and sporulated better on plates containing mono- and disaccharides. Whether this reflects an adaptation to laboratory conditions or represents a strain-specific trait is unknown.

It is known, however, that the carbon source can have an impact on the production of mycotoxins, but the underlying mechanisms are unknown and most likely are not consistent in different fungal genera (82).

In our experiments, cellulose led to a clearly distinct phenotype. The fungus spread extensively, and the resulting flat and weakly sporulating mycelium covered the whole surface of the plates, indicating that the size of the colonies was limited by the size of the plate and could have been larger. This growth phenotype resembled that observed for plates with low amounts of nitrogen, suggesting that both a scarcity of nitrogen and the presence of cellulose as a sole carbon source trigger a hunger phenotype. The low growth efficiency on cellulose plates most likely reflects that the assimilation of cellulose is an elaborate process that requires a multienzymatic system to degrade the polymer and to obtain glucose molecules (83). This limited supply of glucose may also explain the low MT production. Previous data suggested that high cellulose content promotes MT production (22), and *Stachybotrys* is always described as a cellulolytic fungus (81). Our data demonstrated that this might be true in comparison to other fungi, but other carbon sources are better suited for *S. chartarum*, and cultivation on them enables the formation of a dense mycelial network and high MT production.

We also analyzed the impact of the tested nitrogen and carbon sources on production of a set of seven individual MTs. Our data revealed similar patterns for the three strains, and each strain produced RL-2, RE, VJ, SG, SH, and SF but no VA, which is in line with previous studies (37, 54). The observation that an increase or decrease in the total amount of MTs was also largely reflected in the concentrations of the individual MT suggests that the biosynthetic pathways of these compounds are closely linked. This is particularly interesting since such a linkage of the individual MT is expected, although little data exist on the synthesis pathway of these metabolites, and their interpretation relies to a large extent on presumptions. If correct, the current model suggests RE to be the precursor of a biosynthetic process in which VJ, SH, SG, and SF are consecutively formed (84, 85). According to Degenkolb et al. (84), RL-2 takes on a special role as the end product of a side branch that commences at RE, which implies that RL-2 is not a precursor of the satratoxins.

The question of which MT is the most abundantly produced on agar plates or building materials is not answered yet. In previous studies, SH and RE were detected in larger amounts than the other MTs (51, 54). In the present study, RL-2 was the most abundant MT in all samples. Hence, AMM seems to affect the production of mycotoxins in a different way than the other, less defined media. A limitation of the previous studies is that no RL-2 standard was used in these analyses. Instead, the RL-2 concentrations were determined semiquantitatively with the help of other toxins (54). Hinkley and Jarvis (86) produced an RL-2 standard by isolating RL-2 from rice and applying it in their study. Such laboratory-made reagents are prone to variations, for example, due to a different degree of purity. Additionally, it is conceivable that the commercially available standard used in this study may also differ from the one used by Hinkley and Jarvis in the degree of purity.

The measured concentrations of VJ and SF were the lowest of all MTs analyzed. This result must be considered critically since no reference standards were commercially available for VJ and SF, and we had to measure them using a semiquantitative method and determined their concentrations as equivalents of RE and SG (59). It is conceivable that the concentrations could be higher or lower due to a different ionization behavior in LC-MS/MS than RE and SG.

In contrast to MT production, no correlation between colony area, sporulation, and stachybotrylactam (STLAC) production was observed, indicating that the production of STLAC was not tied to colony growth or sporulation and is therefore most likely controlled by different regulatory circuits than MTs. Our results demonstrate that small and nonsporulating colonies were also able to produce high concentrations of STLAC. These data follow previous results described by Jagels et al. (38) where production of PSDs, including STLAC, already takes place at early stages of colony development (i.e., day 3) before any quantities of MTs are detectable and that STLAC is therefore not a suitable marker for fungal growth. Another striking difference between STLAC and MT production is that all nitrogen sources supported a stronger production of STLAC in a manner that was proportional to increasing nitrogen concentrations of up to 250 mg/L. Thus, production was not linked to the phenotype of the corresponding colonies but to the available amount of nitrogen. These findings are in good agreement with previous studies demonstrating that the production of isoindolinone derivatives is favored on nitrogen-rich media (38).

For STLAC production of all three strains, the mono- and disaccharides yielded larger amounts than the two types of starch and in particular cellulose that nearly prevented STLAC production. This correlates well with the previous finding of Ulrich and Schäfer (54) that showed that growth on potato dextrose agar resulted in small amounts of STLAC. However, the finding that the highest STLAC concentrations were detectable on cellulose-containing medium is in contrast to the current results. This discrepancy may reflect the impact of other yet unknown molecules that were present in the cellulose-containing medium used by Ulrich and Schäfer (54).

Since STLAC is a representative of PSDs that are presumed to be immunosuppressive (39, 40), it should be considered that nonsporulating, small colonies may also pose a health risk due to the production of STLAC. Unfortunately, since other PSD toxin standards are not available, it is currently impossible to monitor other PSDs.

### Conclusions.

The analysis of *S. chartarum* genotype S cultures grown on AMM agar plates revealed that various nitrogen and carbon sources distinctly affect morphology and mycotoxin production. NaNO_3_ enabled substantially better growth, resulting in a dense and heavily sporulating mycelium with high MT production if present at concentrations higher than 10 mg N/L. In contrast, a strong nonsupportive effect was observed for ammonium. The different nitrogen sources had a strong impact on growth and MT production, but the different carbon sources that were analyzed also had their influence. Considering the data for growth, sporulation, and MT production, potato starch was the superior and most reliable carbon source for all three strains tested. The results confirmed a positive correlation between sporulation and MT production. In contrast, STLAC production was not linked to colony growth and sporulation, indicating that STLAC and MT production are regulated differently. In view of the supplied compounds, the question of whether well-sporulating or nonsporulating phenotypes are the result of nutrient-induced stress is still a matter of debate. But, the obvious impact of nutrients on secondary metabolism emphasizes the importance of standardization in mycotoxin research of *S. chartarum* and the need for well-defined culture media. AMM was identified as a defined medium, which will be helpful in further investigations of the biosynthetic and metabolic pathways and the regulatory mechanisms that control mycotoxin production in *S*. *chartarum*. The use of AMM will also allow us to determine the impact of other nutritional components, for example, amino acids and salts, in future experiments.

## MATERIALS AND METHODS

### Fungal strains and culture conditions.

In this study, three well-characterized and highly effective MT- and STLAC-producing strains of *S. chartarum* genotype S were analyzed (9, 54), two reference strains (ATCC 34916 and IBT 40293) and one field strain (DSM 114129) that were isolated from animal feed, building material, and culinary herbs, respectively. *S. chartarum* ATCC 34916 was purchased from ATCC (Manassas, VA, USA), *S. chartarum* IBT 40293 was kindly provided by the BioCentrum of the Technical University of Denmark (DTU, Lyngby, Denmark), and *S. chartarum* DSM 114129 was obtained from the Leibniz-Institute DSMZ, German Collection of Microorganisms and Cell Cultures (DSMZ, Braunschweig, Germany).

The fungal strains were preserved long term in sterile 80% glycerol and maintained at −80°C. Working cultures were grown as three-point cultures on potato dextrose agar (VWR Chemicals, Darmstadt, Germany) for 21 days at 25°C and a water activity (a_w_) of 0.98 in the dark. The cultures were checked microscopically for their identity. Five single-spore isolates per strain (≙five biological replicates) were prepared as described previously (57) to ensure that the used strains were pure and to confirm the species, chemotype, and genotype. The respective species identifications were confirmed for all strains by matrix-assisted laser desorption ionization–time of flight mass spectrometry (MALDI-TOF MS) (87). Since *S. chartarum* strains cannot be further differentiated into chemotypes by this method, the production of macrocyclic trichothecenes was analyzed by high-performance liquid chromatography (HPLC) (57) to verify them as chemotype S. Additionally, the genotype of the single-spore isolates was confirmed as *S. chartarum* genotype S by triplex PCR according to Ulrich et al. (10).

Spore suspensions of each strain were prepared from single-spore isolates as described previously (57). Afterward, 10 μL of spore suspension was applied by three-point inoculation on the respective agar plates with the indicated additives (three technical replicates). The plates were analyzed after an incubation period of 21 days at 25°C in the dark. The a_w_ was kept constant at 0.98. The colony areas (cm^2^) were determined for three colonies of three independent agar plates (technical replicates), and the average values were calculated.

The nutrition medium used was Aspergillus minimal medium (AMM) (58); the composition and modifications are shown in Table 1. AMM contains glucose (10 g/L ≙ 4 g C/L) as the carbon source and NaNO_3_ (6 g/L ≙ 988.8 mg N/L) as the nitrogen source. To investigate the impact of the nitrogen source, NaNO_3_ was replaced in some experiments with NH_4_NO_3_ or NH_4_Cl at the indicated concentrations. The amount of the three tested nitrogen sources was adjusted to total concentrations of 1, 5, 10, 25, 50, 250, or 500 mg N/L. In addition, samples without nitrogen addition were tested. All these media contained 10 g/L glucose as the sole carbon source.

**TABLE 1:** Preparation of Aspergillus minimal medium and modifications

Stock solutions		
1. Salt mix (20× stock)	Dissolve the listed salts in 800 mL of distilled water, bring the final volume to 1,000 mL and autoclave.	
	Nitrogen source^*a*^	
	KCl (potassium chloride)	10.4 g
	KH_2_PO_4_ (potassium dihydrogen phosphate)	16.3 g
	K_2_HPO_4_ (dipotassium hydrogen phosphate)	20.9 g
2. MgSO_4_ solution (200× stock)	Dissolve in distilled water, bring the final volume to 500 mL, and autoclave.	
	MgSO_4_ • 7H_2_O (magnesium sulfate heptahydrate)	52.0 g
3. Hunter’s trace element solution (1,000× stock)	**Solution 1**	
	Dissolve the listed salts in 80 mL of distilled water in the following order:	
	FeSO_4_ • 7H_2_O (ferrous sulfate heptahydrate)	1.0 g
	EDTA	10.0 g
	Adjust the pH with KOH pellets until a golden yellow solution is obtained (pH ~ 5.5).	
	**Solution 2**	
	Dissolve the listed salts in 80 mL of distilled water in the following order:	
	ZnSO_4_ • 7H_2_O (zinc sulfate heptahydrate)	4.40 g
	H_3_BO_3_ (boric acid) MnCl_2_ • 4H_2_O (manganese chloride tetrahydrate)	2.20 g 1.00 g
	CoCl_2_ • 6H_2_O (cobalt chloride hexahydrate)	0.32 g
	CuSO_4_ • 5H_2_O (copper sulfate pentahydrate)	0.32 g
	(NH_4_)_6_ Mo_7_O_24_ • 4H_2_O	0.22 g
	(ammoniumheptamolybdate tetrahydrate)	
	Combine solutions 1 and 2 and adjust the pH to 6.5 using KOH pellets first and then KOH solutions of decreasing concentration. Bring the final volume to 200 mL with distilled water.	
Aspergillus minimal medium^*b*^		
	Salt mix (20× stock)	50.0 mL
	MgSO_4_ solution (200× stock)	5.0 mL
	Carbon source^*c*^	
	Hunter’s trace element solution	1.0 mL
	The pH was adjusted to 5.6 and the final volume to 1,000 mL using distilled water. For solid media, add agar (20 g/L) and autoclave for 20 min at 121°C.	

a NaNO_3_, NH_4_NO_3_, or NH_4_Cl were used at concentrations corresponding to total nitrogen concentrations of 1, 5, 10, 25, 50, 250, or 500 mg/L.

b According to Hill and Kafer (58).

c Glucose, fructose, maltose, potato starch, wheat starch, or cellulose was used as the carbon source, and the carbon concentrations were uniformly set to 4 g C/L.

To investigate the influence of the carbon source, glucose was replaced by fructose, maltose, potato starch, wheat starch, or cellulose. In all samples, the carbon concentration was uniformly set to 4 g C/L. Thus, concentrations of 10.0 g/L fructose, 9.5 g/L maltose, 9.0 g/L potato starch, 9.0 g/L wheat starch, and 9.0 g/L cellulose were used. NaNO_3_ (250 mg N/L) was uniformly added as the sole nitrogen source. In addition, strain ATCC 34916 was also tested with NaNO_3_ at a concentration of 25 mg N/L and NH_4_NO_3_ and NH_4_Cl at a concentration of 25 and 250 mg N/L to rule out a variable impact of this nitrogen source in combination with different carbon sources.

Ingredients for preparation of AMM are shown in Table 1, and NaNO_3_ and NH_4_Cl were purchased from Carl Roth GmbH and Co. KG (≥99% *pro analysis* [*p.a.*]; Karlsruhe, Germany). NH_4_NO_3_ was purchased from Sigma-Aldrich (≥99% *p.a.*; St. Louis, MO, USA). Fructose and wheat starch were purchased from Merck Millipore (for biochemistry; Burlington, MA, USA), and maltose, potato starch, and cellulose were purchased from Sigma-Aldrich (≥99% *p.a.*; St. Louis, MO, USA). All media were adjusted to pH 5.6 and sterilized by autoclaving at 121°C for 20 min before use.

### Sample preparation for mycotoxin analysis by LC-MS/MS.

For toxin extraction and purification, each strain was cultured on AMM in triplicate, as described above. Cultures were stored at −20°C until extraction. For toxin extraction, the content of a whole plate was transferred to a mixing bag (Stomacher 80 Biomaster bags, BA6040/STR/DBL strainer double bags, Seward Limited, Worthing, UK). Then, 50 mL of acetonitrile/water (84/16 [vol/vol]) was added (acetonitrile >99.9% and water; HiPerSolv CHROMANORM for HPLC, VWR International GmbH, Darmstadt, Germany), and bags were treated for 5 min in a bag mixer (Stomacher 80 microBiomaster, Seward Limited, Worthing, UK). Subsequently, sample extracts were filtered through a paper filter (Whatman, 595.5, diameter of 185 mm, Maidstone, UK).

Afterward, 3 mL of filtered extract was diluted with 12 mL of water (1:5), and 10 mL of the aqueous toxin extract was purified using solid-phase extraction (SPE) cartridges (Strata-X 33-μm polymeric reversed-phase 500 mg/6 mL, Phenomenex, Aschaffenburg, Germany) as follows: (i) conditioning: 5 mL of methanol (ROTIPURAN >99.9%; Carl Roth GmbH and Co. KG, Karlsruhe, Germany), (ii) equilibration: 5 mL of water (HiPerSolv CHROMANORM for HPLC, VWR International GmbH, Darmstadt, Germany), (iii) loading: 10 mL of diluted aqueous toxin extract, (iv) washing: 10 mL of methanol/water (30/70 [vol/vol]), and (v) elution: 10 mL of methanol (ROTIPURAN >99.9%; Carl Roth GmbH and Co. KG, Karlsruhe, Germany).

The fractions eluted with methanol were evaporated to dryness under a gentle flow of nitrogen and then sent from Ludwig Maximilian University (LMU; Munich, Germany) to Kazimierz Wielki University (Bydgoszcz, Poland) for LC-MS/MS measurements.

The evaporated samples were dissolved in 1 mL of acetonitrile/water (30/70 [vol/vol]) by shaking them for 15 min on a laboratory shaker (2,000 rpm; Multi Reax, Heidolph Instruments, Schwabach, Germany). As a final step, the toxin extracts were filtered through a polyvinylidene difluoride (PVDF) syringe filter (0.20 μm, Ø 13 mm, Macherey-Nagel GmbH and Co. KG, Düren, Germany) and transferred to a 1.5-mL glass thread vial with a cap (VWR International GmbH, Darmstadt, Germany).

### LC-MS/MS measurement and method performance.

LC-MS/MS analysis of all samples was performed using a Shimadzu LC-30AD HPLC equipped with a degassing unit (DGU-20AS), a column oven (CTO-20AC), and an autosampler (SIL-30AC; Duisburg, Germany) and linked to an API 4000 triple quadrupole mass spectrometer (Sciex, Darmstadt, Germany). Analyst 1.6.2 software (Sciex, Darmstadt, Germany) was used for data acquisition and SCIEX OS software 3.0 (Sciex, Darmstadt, Germany) for data analysis.

Chromatography was performed using a Gemini C_18_ 110-Å reversed-phase LC column (150 × 4.6 mm, 5 μm, Phenomenex, Aschaffenburg, Germany) attached to a guard column (SecurityGuard cartridge, Gemini C_18_ 4 × 3.0 mm inside diameter [i.d.], Phenomenex, Aschaffenburg, Germany). The column oven temperature was set to 30°C, and the injection volume was 20 μL. The binary gradient consisting of eluent A (water) and eluent B (methanol; both containing 5 mmol/L ammonium formate and 0.1% formic acid) with a flow rate of 0.75 mL/min was applied as follows: 0 min 10% B, 2 min 10% B, 14 min 97% B, 16 min 97% B, and 16 min 10% B. The column was equilibrated for 4 min under starting conditions before each run. LC-MS-grade water and methanol were purchased from Merck Millipore (Burlington, MA, USA), and LC-MS-grade formic acid and high-purity ammonium formate were purchased from Sigma-Aldrich (St. Louis, MO, USA).

Mass spectrometric measurements were performed using setting parameters as follows: ion spray voltage (electrospray ionization [ESI+]), 5,000 V; temperature, 550°C; nebulizer gas, 50 lb/in^2^; heating gas, 50 lb/in^2^; curtain gas, 25 lb/in^2^; and collision gas (nitrogen, CAD), level 7.

For analyte tuning and qualitative determination, the compounds were identified in multiple reaction monitoring (MRM) mode. Reference standards for roridin E (RE), roridin L-2 (RL-2), satratoxin G (SG), satratoxin H (SH), and stachybotrylactam (STLAC) were purchased from Cayman Chemical (Ann Arbor, MI, USA). Verrucarin J (VJ) and satratoxin F (SF) were purified from rice cultures (using strain ATCC 34916), as described previously (86), and were identified by comparison with substance-specific parameters for identification by LC-MS/MS as described by Ulrich et al. (10). Although verrucarin A (VA) was described to be not produced by *Stachybotrys* species (54, 59), the samples were also analyzed for this mycotoxin. Since no reference standards for VA were commercially available, identification was performed as for VJ and SF. The determined MRM transitions and substance-specific parameters for all compounds used are shown in Table 2.

**TABLE 2:** MRM transitions and substance-specific parameters for the identification of seven macrocyclic trichothecenes and stachybotrylactam by LC-MS/MS

Analyte	Q1 mass (Da)	Q3 mass (Da)	DP^*a*^ (V)	EP^*a*^ (V)	CE^*a*^ (eV)	CXP^*a*^ (V)	Dwell time (ms)
Roridin E	532.36	361.30 113.00	71	10	12 35	26 22	60
Roridin L-2	548.37	249.20 283.10	46	10	21 17	16 20	60
Verrucarin A	520.30	249.10 457.30	71	10	25 19	14 26	40
Verrucarin J	502.30	343.20 249.20	71	10	25 29	20 14	40
Satratoxin G	562.28	249.10 231.10	56	10	19 27	16 16	60
Satratoxin H	546.38	511.30 245.00	56	10	15 27	38 16	60
Satratoxin F	560.30	249.10 231.20	71	10	23 27	14 14	40
Stachybotrylactam	386.28	178.00 150.20	126	10	49 61	10 30	60

a DP, declustering potential; EP, entrance potential; CE, collision energy; CXP, cell exit potential.

Quantification of RE, RL-2, SG, SH, and STLAC was done by a six-point matrix calibration in triplicate for each point (linear regression). Since no reference standards for VA, VJ, and SF were commercially available, they were semiquantitatively determined as equivalents of RE and SG (59). The determined retention times for RE, RL-2, VJ, SG, SH, SF, and STLAC were 14.83 min, 12.66 min, 14.72 min, 13.06 min, 13.30 min, 13.61 min, and 15.28 min, respectively.

To determine matrix suppression effects, solvent and matrix calibrations were done. The matrix effect of AMM was 65% for RE, 40% for RL-2, 63% for SG, 29% for SH, and 25% for STLAC.

The method’s precision was defined by measuring two different concentration levels (20 and 100 ng/mL) multiple times (*n *= 10) and comparing the results with the target value. The precision was between 5.9% and 8.8% for RE, 7.2% and 8.9% for RL-2, 4.1% and 6.7% for SG, 5.0% and 6.6% for SH, and 1.8% and 3.1% for STLAC.

The recovery rate of the extraction method was determined, and the limit of detection (LOD) and limit of quantification (LOQ) were defined. A signal-to-noise (S/N) ratio of 3 was used for LODs, and a signal-to-noise ratio (S/N) of 10 was used for LOQs. The determined LODs and LOQs are shown in Table 3.

**TABLE 3:** Limit of detection and limit of quantification

Target analyte	Aspergillus minimal medium^*a*^	
	LOD (ng/mL)	LOQ (ng/mL)
Roridin E	1.35	4.50
Roridin L-2	0.87	2.90
Satratoxin G	0.33	1.11
Satratoxin H	0.64	2.14
Stachybotrylactam	0.53	1.76

a With glucose (10 g/L) as the carbon source and NaNO_3_ (25 mg N/L) as the nitrogen source.

### Statistical analysis.

Statistical analyses were conducted using the software OriginPro 2021b (64-bit) SR2 (version 9.8.5.212). Comparisons of colony areas and mycotoxin levels observed under various conditions were evaluated for significance by unpaired *t* test (significance level set to a *P* value of <0.05), assuming equal variances for colony areas and unequal variances for mycotoxin levels. Normal distribution was assessed with the Shapiro-Wilk test.

## References

[1] Lombard L, Houbraken J, Decock C, Samson R, Meijer M, Réblová M, Groenewald JZ, Crous PW. 2016. Generic hyper-diversity in *Stachybotriaceae*. Persoonia 36:156–246. doi:10.3767/003158516X691582. 27616791 PMC4988370

[2] Hodgson MJ, Morey P, Leung W-Y, Morrow L, Miller D, Jarvis BB, Robbins H, Halsey JF, Storey E. 1998. Building-associated pulmonary disease from exposure to *Stachybotrys chartarum* and *Aspergillus versicolor*. J Occup Environ Med 40:241–249. doi:10.1097/00043764-199803000-00006. 9531095

[3] Kuhn DM, Ghannoum M. 2003. Indoor mold, toxigenic fungi, and *Stachybotrys chartarum*: infectious disease perspective. Clin Microbiol Rev 16:144–172. doi:10.1128/CMR.16.1.144-172.2003. 12525430 PMC145304

[4] Andersen B, Nielsen KF, Jarvis BB. 2002. Characterization of *Stachybotrys* from water-damaged buildings based on morphology, growth, and metabolite production. Mycologia 94:392–403. 21156510

[5] El-Kady IA, Moubasher MH. 1982. Toxigenicity and toxins of *Stachybotrys* isolates from wheat straw samples in Egypt. Exp Mycol 6:25–30. doi:10.1016/0147-5975(82)90060-3.

[6] Biermaier B, Gottschalk C, Schwaiger K, Gareis M. 2015. Occurrence of *Stachybotrys chartarum* chemotype S in dried culinary herbs. Mycotoxin Res 31:23–32. doi:10.1007/s12550-014-0213-3. 25346283

[7] Li Y, Liu D, Cheng Z, Proksch P, Lin W. 2017. Cytotoxic trichothecene-type sesquiterpenes from the sponge-derived fungus *Stachybotrys chartarum* with tyrosine kinase inhibition. RSC Adv 7:7259–7267. doi:10.1039/C6RA26956G. PMC9066173 35532491

[8] Ibrahim SR, Choudhry H, Asseri AH, Elfaky MA, Mohamed SG, Mohamed GA. 2022. *Stachybotrys chartarum*—a hidden treasure: secondary metabolites, bioactivities, and biotechnological relevance. J Fungi (Basel) 8:504. doi:10.3390/jof8050504. 35628759 PMC9144806

[9] Andersen B, Nielsen KF, Thrane U, Szaro T, Taylor JW, Jarvis BB. 2003. Molecular and phenotypic descriptions of *Stachybotrys chlorohalonata* sp. nov. and two chemotypes of *Stachybotrys chartarum* found in water-damaged buildings. Mycologia 95:1227–1238. doi:10.1080/15572536.2004.11833031. 21149024

[10] Ulrich S, Niessen L, Ekruth J, Schäfer C, Kaltner F, Gottschalk C. 2020. Truncated satratoxin gene clusters in selected isolates of the atranone chemotype of *Stachybotrys chartarum* (Ehrenb.) S. Hughes. Mycotoxin Res 36:83–91. doi:10.1007/s12550-019-00371-x. 31435889 PMC6971138

[11] Dearborn DG, Smith PG, Dahms BB, Allan TM, Sorenson WG, Montana E, Etzel RA. 2002. Clinical profile of 30 infants with acute pulmonary hemorrhage in Cleveland. Pediatrics 110:627–637. doi:10.1542/peds.110.3.627. 12205270

[12] Miller JD, Rand TG, Jarvis BB. 2003. *Stachybotrys chartarum*: cause of human disease or media darling? Med Mycol 41:271–291. doi:10.1080/1369378031000137350. 12964721

[13] Centers for Disease Control and Prevention (CDC). 2000. Update: pulmonary hemorrhage/hemosiderosis among infants—Cleveland, Ohio, 1993–1996. MMWR Morb Mortal Wkly Rep 49:180–184. 11795499

[14] Johanning E, Biagini R, Hull D, Morey P, Jarvis B, Landsbergis P. 1996. Health and immunology study following exposure to toxigenic fungi (*Stachybotrys chartarum*) in a water-damaged office environment. Int Arch Occup Environ Health 68:207–218. doi:10.1007/BF00381430. 8738349

[15] Hintikka E-L. 2004. The role of *Stachybotrys* in the phenomenon known as sick building syndrome. Adv Appl Microbiol 55:155–173. 15350793 10.1016/S0065-2164(04)55005-0

[16] Nikulin M, Reijula K, Jarvis BB, Hintikka EL. 1996. Experimental lung mycotoxicosis in mice induced by *Stachybotrys atra*. Int J Exp Pathol 77:213–218. doi:10.1046/j.1365-2613.1996.9250323.x. 8977373 PMC2691636

[17] Vesper S, Dearborn DG, Yike I, Allan T, Sobolewski J, Hinkley SF, Jarvis BB, Haugland RA. 2000. Evaluation of *Stachybotrys chartarum* in the house of an infant with pulmonary hemorrhage: quantitative assessment before, during, and after remediation. J Urban Health 77:68–85. doi:10.1007/BF02350963. 10741843 PMC3456606

[18] Forgacs J, Carll WT, Herring AS, Hinshaw WR. 1958. Toxicity of *Stachybotrys atra* for animals. Trans N Y Acad Sci 20:787–808. doi:10.1111/j.2164-0947.1958.tb00638.x. 13569556

[19] Schneider DJ, Marasas WF, Dale Kuys JC, Kriek NP, Van Schalkwyk GC. 1979. A field outbreak of suspected stachybotryotoxicosis in sheep. J S Afr Vet Assoc 50:73–81. 575906

[20] Kriek N, Marasas W. 1983. Field outbreak of ovine stachybotryotoxicosis in South Africa, p 279–284. *In* Ueno Y (ed), Thrichothecenes-chemical, biological and toxicological aspects. Elsevier Science Ltd, Amsterdam, the Netherlands.

[21] Semis M, Dadwal SS, Tegtmeier BR, Wilczynski SP, Ito JI, Kalkum M. 2021. First case of invasive *Stachybotrys* sinusitis. Clin Infect Dis 72:1386–1391. doi:10.1093/cid/ciaa231. 32155243

[22] Croft WA, Jarvis BB, Yatawara CS. 1986. Airborne outbreak of trichothecene toxicosis. Atmos Environ 20:549–552. doi:10.1016/0004-6981(86)90096-X.

[23] Gajdusek DC. 1953. Acute infectious hemorrhagic fevers and mycotoxicoses in the union of soviet socialist republics. Literary Licensing, LLC, Whitefish, MT.

[24] Ozegovic L, Pavlovic R, Milosev B. 1971. Toxic dermatitis, conjunctivitis, rhinitis, pharyngitis and laryngitis in fattening cattle and farm workers caused by moulds from contaminated straw (stachybotryotoxicosis?). Veterinaria, Sarajevo 20:263–267.

[25] Grove J. 1988. Non-macrocyclic trichothecenes. Nat Prod Rep 5:187–209. doi:10.1039/np9880500187. 3062504

[26] Grove J. 1993. Macrocyclic trichothecenes. Nat Prod Rep 10:429–448. doi:10.1039/np9931000429. 3062504

[27] Ueno Y. 1984. Toxicological features of T-2 toxin and related trichothecenes. Fundam Appl Toxicol 4:124–132. 10.1016/0272-0590(84)90144-1 6609858

[28] Hanelt M, Gareis M, Kollarczik B. 1994. Cytotoxicity of mycotoxins evaluated by the MTT-cell culture assay. Mycopathologia 128:167–174. doi:10.1007/BF01138479. 7739730

[29] Thompson WL, Wannemacher RW. 1986. Structure-function relationships of 12, 13-epoxytrichothecene mycotoxins in cell culture: comparison to whole animal lethality. Toxicon 24:985–994. doi:10.1016/0041-0101(86)90004-8. 3824405

[30] Wang A, Xu Y, Gao Y, Huang Q, Luo X, An H, Dong J. 2015. Chemical and bioactive diversities of the genera *Stachybotrys* and *Memnoniella* secondary metabolites. Phytochem Rev 14:623–655. doi:10.1007/s11101-014-9365-1.

[31] McLaughlin C, Vaughan M, Campbell I, Wei CM, Stafford M, Hansen B. 1977. Inhibition of protein synthesis by trichothecenes. Mycotoxins in Human and Animal Health 1977:263–273.

[32] Thompson WL, Wannemacher RW. 1990. *In vivo* effects of T-2 mycotoxin on synthesis of proteins and DNA in rat tissues. Toxicol Appl Pharmacol 105:483–491. doi:10.1016/0041-008x(90)90151-j. 2237920

[33] Cundliffe E, Davies JE. 1977. Inhibition of initiation, elongation, and termination of eukaryotic protein synthesis by trichothecene fungal toxins. Antimicrob Agents Chemother 11:491–499. doi:10.1128/AAC.11.3.491. 856003 PMC352012

[34] Islam Z, Harkema JR, Pestka JJ. 2006. Satratoxin G from the black mold *Stachybotrys chartarum* evokes olfactory sensory neuron loss and inflammation in the murine nose and brain. Environ Health Perspect 114:1099–1107. doi:10.1289/ehp.8854. 16835065 PMC1513335

[35] Karunasena E, Larranaga MD, Simoni JS, Douglas DR, Straus DC. 2010. Building-associated neurological damage modeled in human cells: a mechanism of neurotoxic effects by exposure to mycotoxins in the indoor environment. Mycopathologia 170:377–390. doi:10.1007/s11046-010-9330-5. 20549560

[36] Yang B, Long J, Pang X, Lin X, Liao S, Wang J, Zhou X, Li Y, Liu Y. 2021. Structurally diverse polyketides and phenylspirodrimanes from the soft coral-associated fungus *Stachybotrys chartarum* SCSIO41201. J Antibiot (Tokyo) 74:190–198. doi:10.1038/s41429-020-00386-y. 33318621

[37] Jarvis BB, Salemme J, Morais A. 1995. *Stachybotrys* toxins. 1. Nat Toxins 3:10–16. doi:10.1002/nt.2620030104. 7749577

[38] Jagels A, Lindemann V, Ulrich S, Gottschalk C, Cramer B, Hubner F, Gareis M, Humpf HU. 2019. Exploring secondary metabolite profiles of *Stachybotrys* spp. by LC-MS/MS. Toxins (Basel) 11:133. doi:10.3390/toxins11030133. 30818881 PMC6468463

[39] Hong K, Kinoshita T, Miyazaki W, Izawa T, Inoue K. 1979. An anticomplementary agent, K-76 monocarboxylic acid: its site and mechanism of inhibition of the complement activation cascade. J Immunol 122:2418–2423. 448130

[40] Miyazaki W, Tamaoka H, Shinohara M, Kaise H, Izawa T, Nakano Y, Kinoshita T, Hong K, Inoue K. 1980. A complement inhibitor produced by *Stachybotrys complementi*, nov. sp. K‐76, a new species of fungi imperfecti. Microbiol Immunol 24:1091–1108. doi:10.1111/j.1348-0421.1980.tb02914.x. 7219206

[41] Nielsen KF, Holm G, Uttrup L, Nielsen P. 2004. Mould growth on building materials under low water activities. Influence of humidity and temperature on fungal growth and secondary metabolism. Int Biodeterior Biodegradation 54:325–336. doi:10.1016/j.ibiod.2004.05.002.

[42] El-Kady IA, Moubasher MH. 1982. Some cultural conditions that control production of roridin E and Satra H by *Stachybotrys-chartarum*. Cryptogam Mycol 3:151–162. 7202311

[43] Betina V. 1994. Microbial primary and secondary metabolism. Prog Ind Microbiol 30:1–15.

[44] Betina V. 1994. Bioactive secondary metabolite of microorganisms. Prog Ind Microbiol 30:468.

[45] Brzonkalik K, Herrling T, Syldatk C, Neumann A. 2011. The influence of different nitrogen and carbon sources on mycotoxin production in *Alternaria alternata*. Int J Food Microbiol 147:120–126. doi:10.1016/j.ijfoodmicro.2011.03.016. 21496935

[46] Medina Á, Mateo EM, Valle-Algarra FM, Mateo F, Mateo R, Jiménez M. 2008. Influence of nitrogen and carbon sources on the production of ochratoxin A by ochratoxigenic strains of *Aspergillus* spp. isolated from grapes. Int J Food Microbiol 122:93–99. doi:10.1016/j.ijfoodmicro.2007.11.055. 18164776

[47] Mühlencoert E, Mayer I, Zapf MW, Vogel RF, Niessen L. 2004. Production of ochratoxin A by *Aspergillus ochraceus*. Eur J Plant Pathol 110:651–659. doi:10.1023/B:EJPP.0000032404.71695.6b.

[48] Kohut G, Ádám AL, Fazekas B, Hornok L. 2009. N-starvation stress induced FUM gene expression and fumonisin production is mediated via the HOG-type MAPK pathway in *Fusarium proliferatum*. Int J Food Microbiol 130:65–69. doi:10.1016/j.ijfoodmicro.2009.01.002. 19181411

[49] Shim W-B, Woloshuk CP. 1999. Nitrogen repression of fumonisin B1 biosynthesis in *Gibberella fujikuroi*. FEMS Microbiol Lett 177:109–116. doi:10.1111/j.1574-6968.1999.tb13720.x. 10436928

[50] Bata A, Vanyi A, Lepom P, Dashek W, Llewellyn G. 1989. Temperature-dependent toxin production by *Stachybotrys* species, p 393–400. *In* O’Rear CE, Llewellyn GC (ed), Biodeterioration research 2. Springer, New York, NY.

[51] Aleksic B, Bailly S, Draghi M, Pestka JJ, Oswald IP, Robine E, Bailly JD, Lacroix MZ. 2016. Production of four macrocyclic trichothecenes by *Stachybotrys chartarum* during its development on different building materials as measured by UPLC-MS/MS. Build Environ 106:265–273. doi:10.1016/j.buildenv.2016.07.002.

[52] Andersen B, Frisvad JC, Sondergaard I, Rasmussen IS, Larsen LS. 2011. Associations between fungal species and water-damaged building materials. Appl Environ Microbiol 77:4180–4188. doi:10.1128/AEM.02513-10. 21531835 PMC3131638

[53] Jagels A, Stephan F, Ernst S, Lindemann V, Cramer B, Hubner F, Humpf HU. 2020. Artificial vs. natural *Stachybotrys* infestation—comparison of mycotoxin production on various building materials. Indoor Air 30:1268–1282. doi:10.1111/ina.12705. 32510685

[54] Ulrich S, Schäfer C. 2020. Toxin production by *Stachybotrys chartarum* genotype S on different culture media. J Fungi (Basel) 6:159. doi:10.3390/jof6030159. 32887224 PMC7559122

[55] Jarvis B, Hinkley S, Nielsen K. 2000. *Stachybotrys*: an unusual mold associated with water-damaged buildings. Mycotox Res 16:105–108. doi:10.1007/BF02942994. 23605428

[56] Jarvis BB, Lee YW, Comezoglu SN, Yatawara CS. 1986. Trichothecenes produced by *Stachybotrys atra* from Eastern Europe. Appl Environ Microbiol 51:915–918. doi:10.1128/aem.51.5.915-918.1986. 3729393 PMC238987

[57] Tribelhorn K, Twarużek M, Soszczyńska E, Rau J, Baschien C, Straubinger RK, Ebel F, Ulrich S. 2022. Production of satratoxin G and H is tightly linked to sporulation in *Stachybotrys chartarum*. Toxins (Basel) 14:515. doi:10.3390/toxins14080515. 36006177 PMC9413001

[58] Hill TW, Kafer E. 2001. Improved protocols for *Aspergillus* minimal medium: trace element and minimal medium salt stock solutions. Fungal Genet Newsl 48:20–21. doi:10.4148/1941-4765.1173.

[59] Gareis M, Gottschalk C. 2014. *Stachybotrys* spp. and the guttation phenomenon. Mycotoxin Res 30:151–159. doi:10.1007/s12550-014-0193-3. 24619360

[60] Cole RJ, Jarvis BB, Schweikert MA. 2003. Handbook of secondary fungal metabolites, vol 3. Gulf Professional Publishing, Houston, TX.

[61] Calvo AM, Wilson RA, Bok JW, Keller NP. 2002. Relationship between secondary metabolism and fungal development. Microbiol Mol Biol Rev 66:447–459. doi:10.1128/MMBR.66.3.447-459.2002. 12208999 PMC120793

[62] Brodhagen M, Keller NP. 2006. Signalling pathways connecting mycotoxin production and sporulation. Mol Plant Pathol 7:285–301. doi:10.1111/j.1364-3703.2006.00338.x. 20507448

[63] Marzluf G. 1996. Regulation of nitrogen metabolism in mycelial fungi, p 357–368. *In* Brambl R, Marzluf GA (ed), Biochemistry and molecular biology. Springer, New York, NY.

[64] Keller NP, Hohn TM. 1997. Metabolic pathway gene clusters in filamentous fungi. Fungal Genet Biol 21:17–29. doi:10.1006/fgbi.1997.0970. 9126615

[65] Tomsett AB, Garrett RH. 1980. The isolation and characterization of mutants defective in nitrate assimilation in *Neurospora crassa*. Genetics 95:649–660. doi:10.1093/genetics/95.3.649. 6449399 PMC1214252

[66] Johnstone I, McCabe P, Greaves P, Gurr S, Cole G, Brow M, Unkles S, Clutterbuck A, Kinghorn J, Innis M. 1990. Isolation and characterisation of the crnA-niiA-niaD gene cluster for nitrate assimilation in *Aspergillus nidulans*. Gene 90:181–192. doi:10.1016/0378-1119(90)90178-t. 2205530

[67] McQuade AB. 1963. Morphogenesis and nutrition in the *Memnionella-Stachybotrys* group of fungi. J Gen Microbiol 30:429–435. doi:10.1099/00221287-30-3-429. 13932268

[68] ter Schure EG, van Riel NA, Verrips CT. 2000. The role of ammonia metabolism in nitrogen catabolite repression in *Saccharomyces cerevisiae*. FEMS Microbiol Rev 24:67–83. doi:10.1111/j.1574-6976.2000.tb00533.x. 10640599

[69] Pinon R. 1977. Effects of ammonium ions on sporulation of *Saccharomyces cerevisiae*. Exp Cell Res 105:367–378. doi:10.1016/0014-4827(77)90134-3. 321231

[70] Chiu SW, Moore D. 1988. Ammonium ions and glutamine inhibit sporulation of *Coprinus cinereus basidia* assayed *in vitro*. Cell Biol Int Rep 12:519–526. doi:10.1016/0309-1651(88)90038-0. 2902936

[71] Dickinson JR, Dawes IW. 1983. Ammonium ion repression of sporulation in *Saccharomyces cerevisiae*. Microbiology 129:1883–1888. doi:10.1099/00221287-129-6-1883.

[72] van den Berg B, Lister S, Rutherford JC. 2019. Ammonium transceptors: novel regulators of fungal development. PLoS Pathog 15:e1008059. doi:10.1371/journal.ppat.1008059. 31697784 PMC6837285

[73] Brian PW, Curtis P, Hemming H. 1947. Glutinosin: a fungistatic metabolic product of the mould *Metarrhizium glutinosum* S. Pope. Proc R Soc Lond B Biol Sci 135:106–132. doi:10.1098/rspb.1947.0038. 18918877

[74] Patrovsky M, Sinovska K, Branska B, Patakova P. 2019. Effect of initial pH, different nitrogen sources, and cultivation time on the production of yellow or orange *Monascus purpureus* pigments and the mycotoxin citrinin. Food Sci Nutr 7:3494–3500. doi:10.1002/fsn3.1197. 31763000 PMC6848812

[75] Morton A, MacMillan A. 1954. The assimilation of nitrogen from ammonium salts and nitrate by fungi. J Exp Bot 5:232–252. doi:10.1093/jxb/5.2.232.

[76] Takaya N. 2002. Dissimilatory nitrate reduction metabolisms and their control in fungi. J Biosci Bioeng 94:506–510. doi:10.1016/s1389-1723(02)80187-6. 16233342

[77] Kamp A, Høgslund S, Risgaard-Petersen N, Stief P. 2015. Nitrate storage and dissimilatory nitrate reduction by eukaryotic microbes. Front Microbiol 6:1492. doi:10.3389/fmicb.2015.01492. 26734001 PMC4686598

[78] Wiame J-M, Grenson M, Ars HN, Jr. 1985. Nitrogen catabolite repression in yeasts and filamentous fungi. Adv Microb Physiol 26:1–88. 2869649 10.1016/s0065-2911(08)60394-x

[79] Biswas K, Morschhäuser J. 2005. The Mep2p ammonium permease controls nitrogen starvation‐induced filamentous growth in *Candida albicans*. Mol Microbiol 56:649–669. doi:10.1111/j.1365-2958.2005.04576.x. 15819622

[80] Gimeno CJ, Ljungdahl PO, Styles CA, Fink GR. 1992. Unipolar cell divisions in the yeast *S. cerevisiae* lead to filamentous growth: regulation by starvation and RAS. Cell 68:1077–1090. doi:10.1016/0092-8674(92)90079-r. 1547504

[81] Samson R, Houbraken J, Thrane U, Frisvad J, Andersen B. 2010. Food and indoor fungi: CBS-KNAW fungal biodiversity centre, vol 2. CBS-KNAW Fungal Biodiversity Centre, Utrecht, the Netherlands.

[82] Wang W, Liang X, Li Y, Wang P, Keller NP. 2022. Genetic regulation of mycotoxin biosynthesis. J Fungi (Basel) 9:21. doi:10.3390/jof9010021. 36675842 PMC9861139

[83] Saibi W, Abdeljalil S, Gargouri A. 2011. Carbon source directs the differential expression of β-glucosidases in *Stachybotrys microspora*. World J Microbiol Biotechnol 27:1765–1774. doi:10.1007/s11274-010-0634-x.

[84] Degenkolb T, Dieckmann R, Nielsen KF, Gräfenhan T, Theis C, Zafari D, Chaverri P, Ismaiel A, Brückner H, von Döhren H, Thrane U, Petrini O, Samuels GJ. 2008. The *Trichoderma brevicompactum* clade: a separate lineage with new species, new peptaibiotics, and mycotoxins. Mycol Progress 7:177–219. doi:10.1007/s11557-008-0563-3.

[85] McCormick SP, Stanley AM, Stover NA, Alexander NJ. 2011. Trichothecenes: from simple to complex mycotoxins. Toxins (Basel) 3:802–814. doi:10.3390/toxins3070802. 22069741 PMC3202860

[86] Hinkley SF, Jarvis BB. 2000. Chromatographic method for *Stachybotrys* toxins, p 173–194. *In* Trucksess MW, Pohland AE (ed), Mycotoxin protocols, vol 157. Humana Press, Totowa, NJ. 11051002

[87] Ulrich S, Biermaier B, Bader O, Wolf G, Straubinger RK, Didier A, Sperner B, Schwaiger K, Gareis M, Gottschalk C. 2016. Identification of *Stachybotrys* spp. by MALDI-TOF mass spectrometry. Anal Bioanal Chem 408:7565–7581. doi:10.1007/s00216-016-9800-9. 27475444

