# Supplementary material for: A Chemically Defined Medium That Supports Mycotoxin Production by Stachybotrys chartarum Enabled Analysis of the Impact of Nitrogen and Carbon Sources on the Biosynthesis of Macrocyclic Trichothecenes and Stachybotrylactam
Source: Appl Environ Microbiol. 2023 Jun 20;89(7):e00163-23. doi: 10.1128/aem.00163-23 (PMC10370337; doi:10.1128/aem.00163-23)
Supplement: Supplemental file 1 — Supplemental material. Download aem.00163-23-s0001.docx, DOCX file, 6.7 MB [file aem.00163-23-s0001.docx]

**
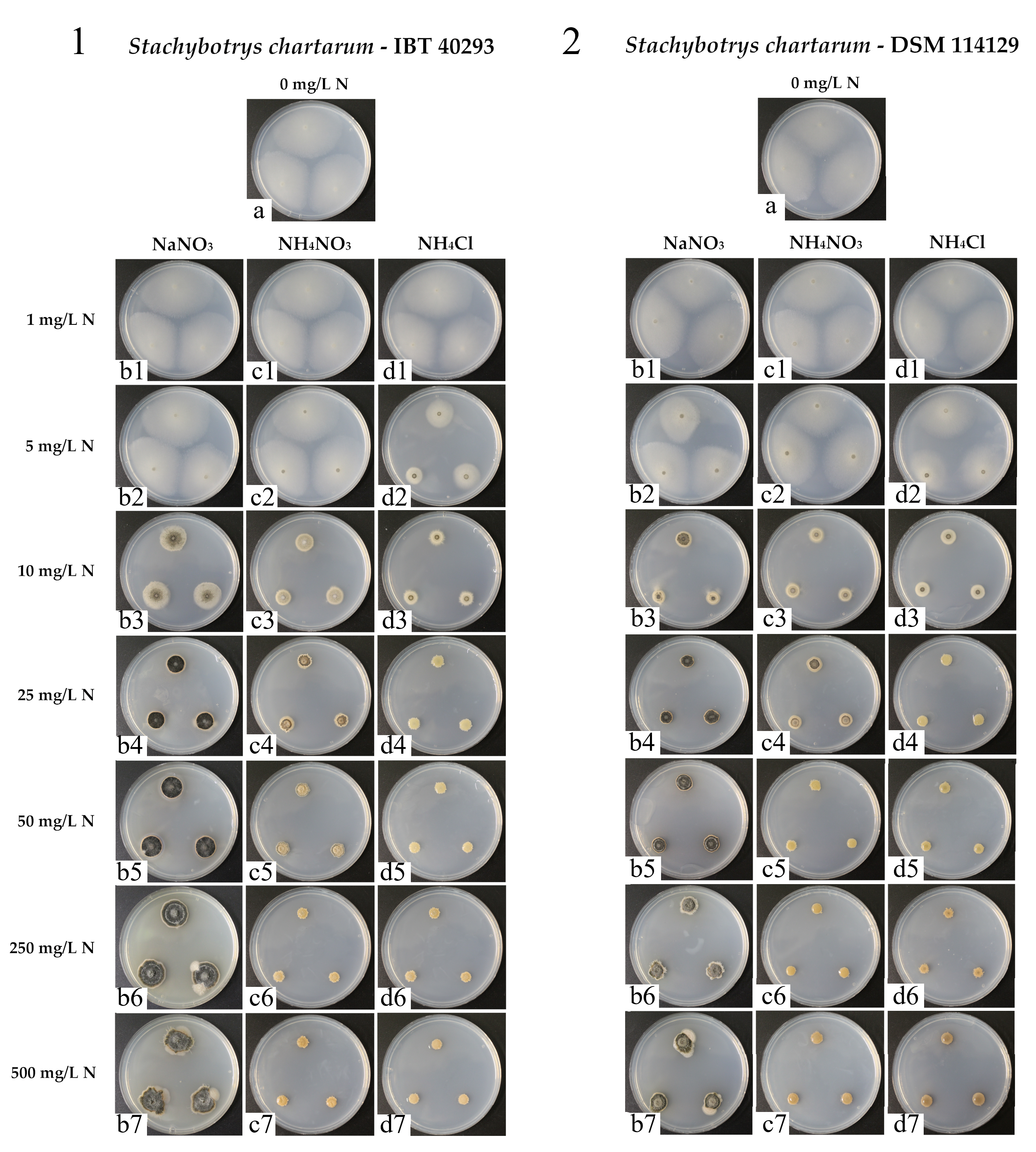
**

**FIG S1** Colonies of *S. chartarum* genotype S strains IBT 40293 (1) and DSM 114129 (2) were grown as three‑point cultures on AMM containing glucose (10 g/L) as sole carbon source. The different panels show cultures without nitrogen addition (1a/2a) or supplemented with either NaNO_3_ (1b1-1b7; 2b1-2b7), NH_4_NO_3_ (1c1‑1c7; 2c1-2c7) or NH_4_Cl (1d1-1d7; 2d1-2d7) at the following nitrogen concentrations: 1 mg/L (1b1/1c1/1d1/2b1/2c1/2d1), 5 mg/L (1b2/1c2/1d2/2b2/2c2/2d2), 10 mg/L (1b3/1c3/1d3/2b3/2c3/2d3), 25 mg/L (1b4/1c4/1d4/2b4/2c4/2d4), 50 mg/L (1b5/1c5/1d5/2b5/2c5/2d5), 250 mg/L (1b6/1c6/1d6/2b6/2c6/2d6), and 500 mg/L (1b7/1c7/1d7/2b7/2c7/2d7). Each image is representative for three parallel cultures per condition.

**TABLE S1** Colony size in cm^2^ measured for cultures of *S. chartarum* genotype S strains ATCC 34916, IBT 40293 and DSM 114129 grown on AMM containing glucose (10 g/L) as sole carbon source and different concentrations of the nitrogen sources NaNO_3_, NH_4_NO_3_, and NH_4_Cl. For representative images of these corresponding cultures compare Figure 1 and Figure S1.

| **Nitrogen source** | **Nitrogen conc.** | **Colony area** | | |
| --- | --- | --- | --- | --- |
|  | **mg/L** | **cm^2^** | | |
|  |  | **ATCC 34916** | **IBT 40293** | **DSM 114129** |
| **w/o nitrogen source** | **0.0** | 58.0 ± 0.8 | 46.3 ± 0.5 | 48.6 ± 1.3 |
| **NaNO_3_** | **1.0**  **5.0**  **10.0**  **25.0**  **50.0**  **250.0**  **500.0** | 58.1 ± 0.3  54.8 ± 1.5  10.4 ± 0.5  6.9 ± 0.2  10.2 ± 0.6  56.8 ± 0.4  57.6 ± 0.2 | 49.0 ± 0.5  44.3 ± 2.7  10.2 ± 0.7  5.2 ± 0.2  6.9 ± 0.2  9.6 ± 1.3  11.6 ± 1.7 | 49.4 ± 2.5  29.0 ± 12.9  3.2 ± 0.4  2.5 ± 0.3  3.9 ± 0.2  4.5 ± 0.2  4.6 ± 0.0 |
| **NH_4_NO_3_** | **1.0**  **5.0**  **10.0**  **25.0**  **50.0**  **250.0**  **500.0** | 58.1 ± 0.2  48.9 ± 0.9  37.7 ± 3.9  5.9 ± 0.6  5.7 ± 0.4  1.9 ± 0.0  2.0 ± 0.1 | 48.2 ± 0.6  43.6 ± 1.5  5.5 ± 1.2  3.3 ± 0.2  2.1 ± 0.2  1.6 ± 0.1  1.4 ± 0.2 | 47.4 ± 2.0  44.0 ± 3.4  3.1 ± 0.3  4.0 ± 1.5  1.2 ± 0.1  1.5 ± 0.5  1.8 ± 0.1 |
| **NH_4_Cl** | **1.0**  **5.0**  **10.0**  **25.0**  **50.0**  **250.0**  **500.0** | 58.3 ± 1.0  40.9 ± 3.5  10.4 ± 0.5  2.2 ± 0.3  1.6 ± 0.1  1.5 ± 0.0  1.4 ± 0.1 | 45.9 ± 1.2  10.2 ± 1.2  4.1 ± 0.2  2.4 ± 0.2  1.8 ± 0.2  1.8 ± 0.1  1.3 ± 0.2 | 47.6 ± 2.4  28.4 ± 3.7  2.5 ± 0.3  2.1 ± 0.3  1.5 ± 0.4  1.3 ± 0.2  1.9 ± 0.2 |

The results are mean values of three replicates ± standard deviation. The pH of the agar plates was adjusted to 5.6. The inoculated media were cultured for 21 days at 25 °C.

**TABLE S2** Colony size in cm^2^ measured for cultures of *S. chartarum* genotype S strains ATCC 34916, IBT 40293 and DSM 114129 grown on AMM containing several carbon sources. The individual carbon sources (4 g carbon per L) were combined with the different nitrogen sources in the indicated concentrations (given as mg nitrogen per L). For representative images of these corresponding cultures compare Figure 2 and Figure 3.

| **Carbon source** | **Nitrogen source** | **Nitrogen conc.** | **Colony**  **area** |
| --- | --- | --- | --- |
|  |  | **mg/L** | **cm^2^** |
| **ATCC 34916** | | | |
| **Glucose** | **NaNO_3_** | **250.0** | 56.8 ± 0.4 |
| **Fructose** | **NaNO_3_** | **25.0**  **250.0** | 5.8 ± 0.2  20.6 ± 3.7 |
|  | **NH_4_NO_3_** | **25.0**  **250.0** | 2.9 ± 0.1  1.9 ± 0.0 |
|  | **NH_4_Cl** | **25.0**  **250.0** | 1.1 ± 0.1  1.5 ± 0.0 |
| **Maltose** | **NaNO_3_** | **25.0**  **250.0** | 10.1 ± 1.0  47.3 ± 0.6 |
|  | **NH_4_NO_3_** | **25.0**  **250.0** | 5.8 ± 0.6  2.6 ± 0.2 |
|  | **NH_4_Cl** | **25.0**  **250.0** | 2.7 ± 0.1  2.8 ± 0.1 |
| **Potato starch** | **NaNO_3_** | **25.0**  **250.0** | 44.2 ± 1.5  40.7 ± 0.5 |
|  | **NH_4_NO_3_** | **25.0**  **250.0** | 38.0 ± 3.2  5.9 ± 0.2 |
|  | **NH_4_Cl** | **25.0**  **250.0** | 7.8 ± 0.5  4.3 ± 0.0 |
| **Wheat starch** | **NaNO_3_** | **25.0**  **250.0** | 12.8 ± 0.3  54.1 ± 1.2 |
|  | **NH_4_NO_3_** | **25.0**  **250.0** | 10.7 ± 0.6  4.3 ± 0.3 |
|  | **NH_4_Cl** | **25.0**  **250.0** | 5.1 ± 0.2  4.1 ± 0.4 |
| **Cellulose** | **NaNO_3_** | **25.0**  **250.0** | 60.8 ± 0.0  60.8 ± 0.0 |
|  | **NH_4_NO_3_** | **25.0**  **250.0** | 60.8 ± 0.0  13.8 ± 1.7 |
|  | **NH_4_Cl** | **25.0**  **250.0** | 29.0 ± 3.6  16.8 ± 1.0 |
|  | | | |
| **IBT 40293** | | | |
| **Glucose** | **NaNO_3_** | **250.0** | 9.6 ± 1.3 |
| **Fructose** | **NaNO_3_** | **250.0** | 6.9 ± 0.2 |
| **Maltose** | **NaNO_3_** | **250.0** | 7.2 ± 0.0 |
| **Potato starch** | **NaNO_3_** | **250.0** | 58.7 ± 0.5 |
| **Wheat starch** | **NaNO_3_** | **250.0** | 50.0 ± 1.0 |
| **Cellulose** | **NaNO_3_** | **250.0** | 60.8 ± 0.0 |
|  | | | |
| **DSM 114129** | | | |
| **Glucose** | **NaNO_3_** | **250.0** | 4.5 ± 0.2 |
| **Fructose** | **NaNO_3_** | **250.0** | 3.0 ± 0.1 |
| **Maltose** | **NaNO_3_** | **250.0** | 5.6 ± 1.0 |
| **Potato starch** | **NaNO_3_** | **250.0** | 27.7 ± 2.5 |
| **Wheat starch** | **NaNO_3_** | **250.0** | 36.5 ± 3.4 |
| **Cellulose** | **NaNO_3_** | **250.0** | 60.8 ± 0.0 |

The results are mean values of three replicates ± standard deviation. The pH of the agar plates was adjusted to 5.6. The inoculated media were cultured for 21 days at 25 °C.

**TABLE S3** Colony size and the amounts of the macrocyclic trichothecenes roridin E (RE), roridin L-2 (RL-2), verrucarin J (VJ), satratoxin G (SG), satratoxin H (SH) and satratoxin F (SF) in ng per agar plate measured for cultures of *S. chartarum* genotype S strains ATCC 34916, IBT 40293 and DSM 114129 grown on AMM containing glucose (10 g/L) as sole carbon source and several nitrogen sources in the indicated concentrations. For representative images of these corresponding cultures compare Figure 1 and Figure S1.

| **Nitrogen source** | **Nitrogen conc.** | **Colony**  **area** | **RE** | **RL-2** | **VJ** | **SG** | **SH** | **SF** | **MT (total)** |
| --- | --- | --- | --- | --- | --- | --- | --- | --- | --- |
|  | **mg/L** | **cm^2^** | **ng/agar plate** | | | | | | |
| **ATCC 34916** | | | | | | | | | |
| **NaNO_3_** | **1.0**  **25.0**  **250.0** | 58.1 ± 0.3  6.9 ± 0.2  56.8 ± 0.4 | 1,904.0 ± 130.0  8,902.7 ± 237.7  5,977.1 ± 1,217.8 | 17,098.6 ± 1,007.4  90,856.8 ± 49,177.7  146,807.5 ± 19,436.6 | 477.8 ± 231.7  1,274.1 ± 659.5  3,991.9 ± 504.6 | 284.0 ± 12.2  11,047.0 ± 461.4  46,162.4 ± 3,114.4 | 488.8 ± 44.2  24,649.6 ± 729.3  47,751.9 ± 2,088.1 | 146.2 ± 10.4  3,848.6 ± 199.2  245.9 ± 41.8 | 20,399.3 ± 1,368.3  140,578.8 ± 48,347.8  250,936.7 ± 21,226.4 |
| **NH_4_NO_3_** | **1.0**  **25.0**  **250.0** | 58.1 ± 0.2  5.9 ± 0.6  1.9 ± 0.0 | 1,463.1 ± 391.4  3,734.4 ± 141.4  n.d. | 10,820.4 ± 4,066.7  40,140.8 ± 3,388.5  n.d. | 252.3 ± 66.5  314.9 ± 6.1  n.d. | 344.0 ± 85.2  1,810.3 ± 35.4  n.d. | 488.3 ± 91.4  6,346.6 ± 269.6  n.d. | 170.5 ± 28.5  962.7 ± 43.7  n.d. | 13,538.6 ± 4,603.0  53,309.7 ± 3,689.2  n.d. |
| **NH_4_Cl** | **1.0**  **25.0**  **250.0** | 58.3 ± 1.0  2.2 ± 0.3  1.5 ± 0.0 | 723.1 ± 49.7  n.d.  n.d. | 4,705.4 ± 756.0  n.d.  n.d. | 98.9 ± 6.8  n.d.  n.d. | 99.3 ± 12.5  n.d.  n.d. | n.d.  n.d.  n.d. | 71.5 ± 17.3  n.d.  n.d. | 5,698.2 ± 781.2  n.d.  n.d. |
| **IBT 40293** | | | | | | | | | |
| **NaNO_3_** | **1.0**  **25.0**  **250.0** | 49.0 ± 0.5  5.2 ± 0.2  9.6 ± 1.3 | 35.7 ± 12.4  4,716.7 ± 261.0  7,129.6 ± 1,180.8 | 410.6 ± 64.0  35,113.8 ± 18,690.5  116,936.6 ± 8,941.4 | n.d.  308.2 ± 22.5  1,163.9 ± 157.3 | n.d.  7,215.2 ± 700.0  12,638.9 ± 2,224.4 | n.d.  14,611.7 ± 1,083.3  27,210.2 ± 3,102.3 | n.d.  2,323.8 ± 271.7  2,340.8 ± 207.8 | 446.3 ± 52.6  64,289.5 ± 19,647.0  167,420.1 ± 15,645.0 |
| **NH_4_NO_3_** | **1.0**  **25.0**  **250.0** | 48.2 ± 0.6  3.3 ± 0.2  1.6 ± 0.1 | 73.9 ± 20.1  971.8 ± 432.0  n.d. | 673.9 ± 219.4  7,429.6 ± 2,990.6  n.d. | n.d.  14.1 ± 5.6  n.d. | n.d.  397.1 ± 224.8  n.d. | n.d.  791.9 ± 533.1  n.d. | n.d.  166.0 ± 46.9  n.d. | 747.9 ± 239.0  9,770.3 ± 4,209.6  n.d. |
| **NH_4_Cl** | **1.0**  **25.0**  **250.0** | 45.9 ± 1.2  2.4 ± 0.2  1.8 ± 0.1 | n.d.  n.d.  n.d. | 266.9 ± 109.7  n.d.  n.d. | n.d.  n.d.  n.d. | n.d.  n.d.  n.d. | n.d.  n.d.  n.d. | n.d.  n.d.  n.d. | 266.9 ± 109.7  n.d.  n.d. |
| **DSM 114129** | | | | | | | | | |
| **NaNO_3_** | **1.0**  **25.0**  **250.0** | 49.4 ± 2.5  2.5 ± 0.3  4.5 ± 0.2 | 1,005.9 ± 13.1  3,787.0 ± 112.2  4,334.9 ± 279.7 | 6,098.6 ± 199.7  21,366.2 ± 1,248.9  45,316.9 ± 4,060.3 | 29.4 ± 5.6  276.4 ± 17.5  488.7 ± 52.2 | 73.4 ± 5.8  1,682.1 ± 165.5  2,873.7 ± 426.7 | n.d.  6,329.5 ± 93.0  9,814.4 ± 1,227.0 | 92.9 ± 10.0  865.4 ± 68.8  689.8 ± 61.5 | 7,300.2 ± 195.5  34,306.6 ± 1,025.5  63,518.4 ± 5,950.8 |
| **NH_4_NO_3_** | **1.0**  **25.0**  **250.0** | 47.4 ± 2.0  4.0 ± 1.5  1.5 ± 0.5 | 1,167.6 ± 120.5  1,261.3 ± 374.1  n.d. | 6,380.3 ± 1,746.1  7,174.9 ± 2,671.7  n.d. | 33.4 ± 9.8  23.4 ± 7.6  n.d. | 89.9 ± 4.5  274.3 ± 110.4  n.d. | n.d.  476.2 ± 390. 8  n.d. | 100.9 ± 4.5  255.8 ± 56.8  n.d. | 7,771.8 ± 1,831.9  9,465.8 ± 3,547.8  n.d. |
| **NH_4_Cl** | **1.0**  **25.0**  **250.0** | 47.6 ± 2.4  2.1 ± 0.3  1.3 ± 0.2 | 756.6 ± 140.4  n.d.  n.d. | 4,543.4 ± 383.3  n.d.  n.d. | 23.3 ± 3.7  n.d.  n.d. | n.d.  n.d.  n.d. | n.d.  n.d.  n.d. | n.d.  n.d.  n.d. | 5,323.4 ± 455.4  n.d.  n.d. |

The results are mean values of three replicates ± standard deviation. The pH of the agar plates was adjusted to 5.6. The inoculated media were cultured for 21 days at 25 °C; n.d.: not detectable or rather under the limit of detection (LOD, signal-to-noise ratio (S/N) ≥ 3).

**TABLE S4** Colony size and **t**he amounts of the macrocyclic trichothecenes roridin E (RE), roridin L-2 (RL-2), verrucarin J (VJ), satratoxin G (SG), satratoxin H (SH) and satratoxin F (SF) in ng per agar plate measured for cultures of *S. chartarum* genotype S strains ATCC 34916, IBT 40293 and DSM 114129 grown on AMM containing several carbon sources. The carbon sources were combined with different nitrogen sources in two concentrations (given as mg nitrogen per L). For representative images of these corresponding cultures compare Figure 2 and Figure 3.

| **Carbon source** | **Nitrogen source** | **Nitrogen conc.** | **Colony**  **area** | **RE** | **RL-2** | **VJ** | **SG** | **SH** | **SF** | **MT (total)** |
| --- | --- | --- | --- | --- | --- | --- | --- | --- | --- | --- |
|  |  | **mg/L** | **cm^2^** | **ng/agar plate** | | | | | | |
| **ATCC 34916** | | | | | | | | | | |
| **Glucose** | **NaNO_3_** | **250.0** | 56.8 ± 0.4 | 5,977.1 ± 1217.8 | 146,807.5 ± 19,436.6 | 3,991.9 ± 504.6 | 46,162.4 ± 3,114.4 | 47,751.9 ± 2,088.1 | 245.9 ± 41.8 | 250,936.7 ± 21,226.4 |
| **Fructose** | **NaNO_3_** | **25.0**  **250.0** | 5.8 ± 0.2  20.6 ± 3.7 | 4,143.3 ± 107.4  3,396.5 ± 198.5 | 25,150.2 ± 2,894.5  41,217.1 ± 1,2667.6 | 441.0 ± 47.0  1,714.6 ± 449.2 | 10,279.9 ± 307.8  9,758.5 ± 584.7 | 14,589.0 ± 384.7  17,731.1 ± 898.5 | 3,895.8 ± 118.2  56.4 ± 23.7 | 58,499.2 ± 2,459.5  73,874.2 ± 12,057.3 |
|  | **NH_4_NO_3_** | **25.0**  **250.0** | 2.9 ± 0.1  1.9 ± 0.0 | 1,560.1 ± 42.5  n.d. | 9,703.1 ± 1,996.9  n.d. | 77.2 ± 5.9  n.d. | 1,490.0 ± 101.1  n.d. | 2,674.4 ± 173.1  n.d. | 925.6 ± 33.8  n.d. | 16,430.3 ± 1,800.1  n.d. |
|  | **NH_4_Cl** | **25.0**  **250.0** | 1.1 ± 0.1  1.5 ± 0.0 | n.d.  n.d. | n.d.  n.d. | n.d.  n.d. | n.d.  n.d. | n.d.  n.d. | n.d.  n.d. | n.d.  n.d. |
| **Maltose** | **NaNO_3_** | **25.0**  **250.0** | 10.1 ± 1.0  47.3 ± 0.6 | 3,952.0 ± 646.1  3,436.2 ± 181.0 | 42,137.3 ± 12,685.9  71,525.8 ± 7,113.4 | 869.2 ± 409.6  2,977.3 ± 29.6 | 4,767.3 ± 853.4  39,555.6 ± 931.6 | 8,589.0 ± 1,428.6  35,566.3 ± 221.0 | 1,768.3 ± 195.5  173.3 ± 56.6 | 62,083.2 ± 13,978.2  153,234.4 ± 7,201.6 |
|  | **NH_4_NO_3_** | **25.0**  **250.0** | 5.8 ± 0.6  2.6 ± 0.2 | 2,115.8 ± 171.4  n.d. | 17,247.7 ± 2,064.4  n.d. | 158.7 ± 16.5  n.d. | 1,241.7 ± 150.0  n.d. | 2,896.2 ± 493.5  n.d. | 747.5 ± 90.5  n.d. | 24,407.5 ± 2,812.4  n.d. |
|  | **NH_4_Cl** | **25.0**  **250.0** | 2.7 ± 0.1  2.8 ± 0.1 | 208.6 ± 214.5  n.d. | 2,022.7 ± 2,091.8  n.d. | n.d.  n.d. | n.d.  n.d. | n.d.  n.d. | n.d.  n.d. | 2,231.3 ± 2,305.7  n.d. |
| **Potato starch** | **NaNO_3_** | **25.0**  **250.0** | 44.2 ± 1.5  40.7 ± 0.5 | 9,111.4 ± 193.2  21,558.9 ± 1,079.9 | 119,248.8 ± 9,969.5  238,544.6 ± 12,237.7 | 1,230.7 ± 70.8  3,106.6 ± 161.2 | 19,619.7 ± 696.6  51,636.8 ± 4,121.6 | 26,448.9 ± 1,384.0  46,202.7 ± 2,866.9 | 5,332.2 ± 210.7  8,600.8 ± 1,063.7 | 180,991.7 ± 12,310.3  369,650.3 ± 17,550.5 |
|  | **NH_4_NO_3_** | **25.0**  **250.0** | 38.0 ± 3.2  5.9 ± 0.2 | 5,345.5 ± 177.3  n.d. | 30,495.3 ± 933.2  n.d. | 472.6 ± 19.4  n.d. | 18,589.9 ± 4,075.5  n.d. | 22,460.2 ± 911.1  n.d. | 5,134.8 ± 145.6  n.d. | 80,154.2 ± 2,006.9  n.d. |
|  | **NH_4_Cl** | **25.0**  **250.0** | 7.8 ± 0.5  4.3 ± 0.0 | 33.1 ± 27.2  n.d. | 806.6 ± 311.6  n.d. | n.d.  n.d. | 118.4 ± 106.6  n.d. | n.d.  n.d. | n.d.  n.d. | 958.1 ± 438.6  n.d. |
| **Wheat starch** | **NaNO_3_** | **25.0**  **250.0** | 12.8 ± 0.3  54.1 ± 1.2 | 3,861.1 ± 646.0  5,110.6 ± 137.7 | 28,909.6 ± 5,589.9  67,570.4 ± 11,466.1 | 527.9 ± 71.2  3,048.4 ± 338.8 | 20,529.9 ± 3,055.2  44,891.0 ± 2,855.8 | 19,856.1 ± 2,786.5  35,986.7 ± 1,136.9 | 5,064.1 ± 694.9  234.3 ± 130.7 | 78,748.8 ± 12,759.6  156,841.4 ± 14,065.5 |
|  | **NH_4_NO_3_** | **25.0**  **250.0** | 10.7 ± 0.6  4.3 ± 0.3 | 1,152.3 ± 168.6  n.d. | 9,377.9 ± 409.2  n.d. | 121.6 ± 15.2  n.d. | 4,783.8 ± 902.5  n.d. | 4,744.1 ± 571.3  n.d. | 1,099.4 ± 233.4  n.d. | 21,279.1 ± 2,283.1  n.d. |
|  | **NH_4_Cl** | **25.0**  **250.0** | 5.1 ± 0.2  4.1 ± 0.4 | n.d.  n.d. | n.d.  n.d. | n.d.  n.d. | n.d.  n.d. | n.d.  n.d. | n.d.  n.d. | n.d.  n.d. |
| **Cellulose** | **NaNO_3_** | **25.0**  **250.0** | 60.8 ± 0.0  60.8 ± 0.0 | 4,066.3 ± 612.8  4,854.9 ± 484.5 | 48,603.3 ± 6,759.5  28,761.7 ± 11,033.5 | 762.0 ± 104.1  2,344.0 ± 590.5 | 21,850.4 ± 1,669.8  39,132.5 ± 9,631.9 | 22,786.0 ± 2,617.1  36,560.6 ± 8,185.1 | 3,169.8 ± 1,110.9  89.6 ± 42.0 | 101,237.8 ± 11,671.9  111,743.3 ± 29,859.1 |
|  | **NH_4_NO_3_** | **25.0**  **250.0** | 60.8 ± 0.0  13.8 ± 1.7 | 172.4 ± 11.2  n.d. | 2,876.1 ± 266.0  n.d. | 88.9 ± 11.3  n.d. | 987.8 ± 37.5  n.d. | 501.2 ± 302.0  n.d. | 58.8 ± 7.0  n.d. | 4,685.2 ± 603.0  n.d. |
|  | **NH_4_Cl** | **25.0**  **250.0** | 29.0 ± 3.6  16.8 ± 1.0 | 11.1 ± 0.0  n.d. | 654.1 ± 193.8  n.d. | n.d.  n.d. | 293.8 ± 72.0  n.d. | n.d.  n.d. | n.d.  n.d. | 947.9 ± 265.7  n.d. |
|  | | | | | | | | | | |
| **IBT 40293** | | | | | | | | | | |
| **Glucose** | **NaNO_3_** | **250.0** | 9.6 ± 1.3 | 7,129.6 ± 1,180.8 | 116,936.6 ± 8,941.4 | 1,163.9 ± 157.3 | 12,638.9 ± 2,224.4 | 27,210.2 ± 3,102.3 | 2,340.8 ± 207.8 | 167,420.1 ± 15,645.0 |
| **Fructose** | **NaNO_3_** | **250.0** | 6.9 ± 0.2 | 3,418.0 ± 307.2 | 58,838.0 ± 7,694.2 | 396.0 ± 57.7 | 5,324.6 ± 728.8 | 12,602.3 ± 1,811.7 | 949.2 ± 242.5 | 81,528.1 ± 10,424.5 |
| **Maltose** | **NaNO_3_** | **250.0** | 7.2 ± 0.0 | 1,509.0 ± 139.0 | 12,130.3 ± 10,737.8 | 144.6 ± 72.8 | 1,504.5 ± 387.7 | 2,753.2 ± 693.2 | 112.4 ± 24.4 | 18,154.0 ± 9,504.0 |
| **Potato starch** | **NaNO_3_** | **250.0** | 58.7 ± 0.5 | 28,025.2 ± 2,408.0 | 347,676.1 ± 23,408.6 | 3,425.1 ± 625.7 | 107,991.5 ± 6,016.2 | 133,958.3 ± 6,577.5 | 11,571.6 ± 1,110.7 | 632,647.7 ± 32,854.1 |
| **Wheat starch** | **NaNO_3_** | **250.0** | 50.0 ± 1.0 | 7,335.5 ± 564.2 | 100,410.8 ± 5,656.8 | 2,171.8 ± 246.8 | 53,450.9 ± 1,176.7 | 54,456.4 ± 3,283.6 | 302.4 ± 94.5 | 218,127.8 ± 3,038.7 |
| **Cellulose** | **NaNO_3_** | **250.0** | 60.8 ± 0.0 | 9,035.4 ± 1,486.3 | 44,062.2 ± 11,486.0 | 1,849.3 ± 266.2 | 35,779.9 ± 5,632.6 | 47,871.2 ± 8,545.8 | 77.8 ± 13.5 | 138,675.7 ± 25,655.7 |
|  | | | | | | | | | | |
| **DSM 114129** | | | | | | | | | | |
| **Glucose** | **NaNO_3_** | **250.0** | 4.5 ± 0.2 | 4,334.9 ± 279.7 | 45,316.9 ± 4,060.3 | 488.7 ± 52.2 | 2,873.7 ± 426.7 | 9,814.4 ± 1,227.0 | 689.8 ± 61.5 | 63,518.4 ± 5,950.8 |
| **Fructose** | **NaNO_3_** | **250.0** | 3.0 ± 0.1 | 261.5 ± 33.1 | 3,498.8 ± 354.0 | 10.3 ± 0.6 | n.d. | n.d. | n.d. | 3,770.6 ± 380.4 |
| **Maltose** | **NaNO_3_** | **250.0** | 5.6 ± 1.0 | 1,709.4 ± 386.9 | 15,556.3 ± 2,744.3 | 392.9 ± 300.1 | 3,978.0 ± 2,850.3 | 5,986.9 ± 2,680.4 | 206.2 ± 78.0 | 27,829.7 ± 7,180.9 |
| **Potato starch** | **NaNO_3_** | **250.0** | 27.7 ± 2.5 | 14,543.9 ± 1,137.3 | 179,331.0 ± 21,193.7 | 2,793.8 ± 931.6 | 51,613.2 ± 8,327.7 | 76,458.3 ± 8,046.6 | 7,617.7 ± 317.5 | 332,358.0 ± 39,530.4 |
| **Wheat starch** | **NaNO_3_** | **250.0** | 36.5 ± 3.4 | 17,143.4 ± 2,394.0 | 162,112.7 ± 67,668.6 | 5,639.2 ± 1,930.6 | 50,957.3 ± 5,897.6 | 82,594.7 ± 10,710.8 | 3,210.2 ± 1,897.9 | 321,657.5 ± 84,045.9 |
| **Cellulose** | **NaNO_3_** | **250.0** | 60.8 ± 0.0 | 5,278.2 ± 1,162.8 | 13,217.1 ± 4,669.5 | 1,140.3 ± 442.0 | 20,138.9 ± 7,032.4 | 31,365.5 ± 5,826.9 | 39.1 ± 5.1 | 71,179.1 ± 19,120.8 |

The results are mean values of three replicates ± standard deviation. The pH of the agar plates was adjusted to 5.6. The inoculated media were cultured for 21 days at 25 °C; n.d.: not detectable or rather under the LOD (signal-to-noise ratio (S/N) ≥ 3).

**TABLE S5** Colony size and **t**he amount of stachybotrylactam in ng per agar plate measured for cultures of *S. chartarum* genotype S strains ATCC 34916, IBT 40293 and DSM 114129 grown on AMM containing glucose (10 g/L) as sole carbon source and different concentrations of three nitrogen sources. For representative images of these corresponding cultures compare Figure 1 and Figure S1.

| **Nitrogen source** | **Nitrogen conc.** | **Colony area** | **Stachybotrylactam** |
| --- | --- | --- | --- |
|  | **mg/L** | **cm^2^** | **ng/agar plate** |
| **ATCC 34916** | | | |
| **NaNO_3_** | **1.0**  **25.0**  **250.0** | 58.1 ± 0.3  6.9 ± 0.2  56.8 ± 0.4 | 4,140.6 ± 481.2  8,335.5 ± 719.1  231,880.3 ± 31,624.9 |
| **NH_4_NO_3_** | **1.0**  **25.0**  **250.0** | 58.1 ± 0.2  5.9 ± 0.6  1.9 ± 0.0 | 2,852.4 ± 751.9  14,747.9 ± 1,938.0  15,085.5 ± 2,438.3 |
| **NH_4_Cl** | **1.0**  **25.0**  **250.0** | 58.3 ± 1.0  2.2 ± 0.3  1.5 ± 0.0 | 10,194.2 ± 7,282.1  474.4 ± 54.6  17,376.1 ± 3,449.3 |
|  | | | |
| **IBT 40293** | | | |
| **NaNO_3_** | **1.0**  **25.0**  **250.0** | 49.0 ± 0.5  5.2 ± 0.2  9.6 ± 1.3 | n.d.  15,722.2 ± 487.2  50,429.5 ± 11,147.4 |
| **NH_4_NO_3_** | **1.0**  **25.0**  **250.0** | 48.2 ± 0.6  3.3 ± 0.2  1.6 ± 0.1 | n.d.  25,190.2 ± 2,029.1  33,465.8 ± 7,609.4 |
| **NH_4_Cl** | **1.0**  **25.0**  **250.0** | 45.9 ± 1.2  2.4 ± 0.2  1.8 ± 0.1 | n.d.  2,306.4 ± 354.1  42,388.9 ± 5,665.4 |
|  | | | |
| **DSM 114129** | | | |
| **NaNO_3_** | **1.0**  **25.0**  **250.0** | 49.4 ± 2.5  2.5 ± 0.3  4.5 ± 0.2 | 478.8 ± 113.7  14,987.2 ± 1,323.9  41,517.1 ± 2,859.7 |
| **NH_4_NO_3_** | **1.0**  **25.0**  **250.0** | 47.4 ± 2.0  4.0 ± 1.5  1.5 ± 0.5 | n.d.  26,617.5 ± 2,768.4  88,547.0 ± 9,863.5 |
| **NH_4_Cl** | **1.0**  **25.0**  **250.0** | 47.6 ± 2.4  2.1 ± 0.3  1.3 ± 0.2 | 523.7 ± 79.6  16,000.0 ± 1,224.2  83,953.0 ± 2,722.9 |

The results are mean values of three replicates ± standard deviation. The pH of the agar plates was adjusted to 5.6. The inoculated media were cultured for 21 days at 25 °C; n.d.: not detectable or rather under the LOD (signal-to-noise ratio (S/N) ≥ 3).

**TABLE S6** Colony size and the amount of stachybotrylactam in ng per agar plate measured for cultures of *S. chartarum* genotype S strains ATCC 34916, IBT 40293 and DSM 114129 grown on AMM containing six different carbon sources in combination with three different nitrogen sources. For representative images of these corresponding cultures compare Figure 2 and Figure 3.

| **Carbon source** | **Nitrogen source** | **Nitrogen conc.** | **Colony area** | **Stachybotrylactam** |
| --- | --- | --- | --- | --- |
|  |  | **mg/L** | **cm^2^** | **ng/agar plate** |
| **ATCC 34916** | | | | |
| **Glucose** | **NaNO_3_** | **250.0** | 56.8 ± 0.4 | 231,880.3 ± 31,624.9 |
| **Fructose** | **NaNO_3_** | **25.0**  **250.0** | 5.8 ± 0.2  20.6 ± 3.7 | 12,096.2 ± 674.4  237,414.5 ± 24,817.7 |
|  | **NH_4_NO_3_** | **25.0**  **250.0** | 2.9 ± 0.1  1.9 ± 0.0 | 22,589.7 ± 8,035.5  14,243.6 ± 2,048.1 |
|  | **NH_4_Cl** | **25.0**  **250.0** | 1.1 ± 0.1  1.5 ± 0.0 | 524.2 ± 54.1  10,433.8 ± 470.3 |
| **Maltose** | **NaNO_3_** | **25.0**  **250.0** | 10.1 ± 1.0  47.3 ± 0.6 | 6,291.9 ± 439.2  175,598.3 ± 7904.7 |
|  | **NH_4_NO_3_** | **25.0**  **250.0** | 5.8 ± 0.6  2.6 ± 0.2 | 19,297.0 ± 1,500.1  13,096.2 ± 1,605.8 |
|  | **NH_4_Cl** | **25.0**  **250.0** | 2.7 ± 0.1  2.8 ± 0.1 | 1,958.5 ± 2,104.4  14,453.0 ± 1,768.6 |
| **Potato starch** | **NaNO_3_** | **25.0**  **250.0** | 44.2 ± 1.5  40.7 ± 0.5 | 6,161.3 ± 304.6  52,617.5 ± 7,695.3 |
|  | **NH_4_NO_3_** | **25.0**  **250.0** | 38.0 ± 3.2  5.9 ± 0.2 | 9,884.6 ± 1,881.2  11,126.1 ± 3,647.7 |
|  | **NH_4_Cl** | **25.0**  **250.0** | 7.8 ± 0.5  4.3 ± 0.0 | 7,794.0 ± 10,014.2  4,138.2 ± 514.3 |
| **Wheat starch** | **NaNO_3_** | **25.0**  **250.0** | 12.8 ± 0.3  54.1 ± 1.2 | 11,326.9 ± 1,237.1  103,760.7 ± 3,645.6 |
|  | **NH_4_NO_3_** | **25.0**  **250.0** | 10.7 ± 0.6  4.3 ± 0.3 | 22,348.3 ± 2,258.2  7,309.6 ± 1,839.6 |
|  | **NH_4_Cl** | **25.0**  **250.0** | 5.1 ± 0.2  4.1 ± 0.4 | 417.1 ± 110.0  13,487.2 ± 3,077.4 |
| **Cellulose** | **NaNO_3_** | **25.0**  **250.0** | 60.8 ± 0.0  60.8 ± 0.0 | 7,185.9 ± 2,076.0  6,764.3 ± 874.0 |
|  | **NH_4_NO_3_** | **25.0**  **250.0** | 60.8 ± 0.0  13.8 ± 1.7 | 909.8 ± 240.5  1,686.7 ± 1,918.3 |
|  | **NH_4_Cl** | **25.0**  **250.0** | 29.0 ± 3.6  16.8 ± 1.0 | n.d.  222.9 ± 222.7 |
|  | | | | |
| **IBT 40293** | | | | |
| **Glucose** | **NaNO_3_** | **250.0** | 9.6 ± 1.3 | 50,429.5 ± 11,147.4 |
| **Fructose** | **NaNO_3_** | **250.0** | 6.9 ± 0.2 | 44,694.4 ± 11,073.7 |
| **Maltose** | **NaNO_3_** | **250.0** | 7.2 ± 0.0 | 24,335.5 ± 1,543.9 |
| **Potato starch** | **NaNO_3_** | **250.0** | 58.7 ± 0.5 | 55,173.1 ± 10,563.8 |
| **Wheat starch** | **NaNO_3_** | **250.0** | 50.0 ± 1.0 | 126,559.8 ± 11,530.7 |
| **Cellulose** | **NaNO_3_** | **250.0** | 60.8 ± 0.0 | 4,252.1 ± 1,333.4 |
|  | | | | |
| **DSM 114129** | | | | |
| **Glucose** | **NaNO_3_** | **250.0** | 4.5 ± 0.2 | 41,517.1 ± 2,859.7 |
| **Fructose** | **NaNO_3_** | **250.0** | 3.0 ± 0.1 | 22,160.3 ± 1,162.5 |
| **Maltose** | **NaNO_3_** | **250.0** | 5.6 ± 1.0 | 39,425.2 ± 7,798.7 |
| **Potato starch** | **NaNO_3_** | **250.0** | 27.7 ± 2.5 | 25,312.0 ± 7,747.9 |
| **Wheat starch** | **NaNO_3_** | **250.0** | 36.5 ± 3.4 | 51,976.5 ± 14,229.6 |
| **Cellulose** | **NaNO_3_** | **250.0** | 60.8 ± 0.0 | 2,775.6 ± 985.9 |

The results are mean values of three replicates ± standard deviation. The different carbon sources were normalized to 4 g carbon per L. The pH of the agar plates was adjusted to 5.6. The inoculated media were cultured for 21 days at 25 °C; n.d.: not detectable or rather under the LOD (signal-to-noise ratio (S/N) ≥ 3).
